# Chemical alternatives assessment of different flame retardants – A case study including multi-walled carbon nanotubes as synergist^☆^

**DOI:** 10.1016/j.envint.2016.12.017

**Published:** 2017-04

**Authors:** Karin Aschberger, Ivana Campia, Laia Quiros Pesudo, Anita Radovnikovic, Vittorio Reina

**Affiliations:** European Commission, Joint Research Centre (JRC), Directorate for Health, Consumers and Reference Materials, Via E. Fermi 2749, I-21027 Ispra, VA, Italy

**Keywords:** Chemical alternatives assessment, Flame retardant, Hazard, Risk, Multi-walled carbon nanotubes

## Abstract

Flame retardants (FRs) are a diverse group of chemicals used as additives in a wide range of products to inhibit, suppress, or delay ignition and to prevent the spread of fire. Halogenated FRs (HFRs) are widely used because of their low impact on other material properties and the low loading levels necessary to meet the required flame retardancy. Health and environmental hazards associated with some halogenated FRs have driven research for identifying safer alternatives. A variety of halogen-free FRs are available on the market, including organic (phosphorus and nitrogen based chemicals) and inorganic (metals) materials. Multi-walled carbon nanotubes (MWCNT) have been demonstrated to act as an effective/synergistic co-additive in some FR applications and could thereby contribute to reducing the loading of FRs in products and improving their performance.

As part of the FP7 project DEROCA we carried out a chemical alternatives assessment (CAA). This is a methodology for identifying, comparing and selecting safer alternatives to chemicals of concern based on criteria for categorising human and environmental toxicity as well as environmental fate. In the project we assessed the hazard data of different halogen-free FRs to be applied in 5 industrial and consumer products and here we present the results for MWCNT, aluminium diethylphosphinate, aluminium trihydroxide, *N*-alkoxy hindered amines and red phosphorus compared to the HFR decabromodiphenylether. We consulted the REACH guidance, the criteria of the U.S.-EPA Design for Environment (DfE) and the GreenScreen® Assessment to assess and compare intrinsic properties affecting the hazard potential.

A comparison/ranking of exposure reference values such as Derived No Effect Levels (DNELs) showed that FRs of concern are not identified by a low DNEL. A comparison based on hazard designations according to the U.S.-EPA DfE and GreenScreen® for human health endpoints, aquatic toxicity and environmental fate showed that the major differences between FRs of concern and their proposed alternatives are the potential for bioaccumulation and CMR (carcinogenic, mutagenic or reprotoxic) effects. As most alternatives are inorganic chemicals, persistence (alone) is not a suitable criterion.

From our experiences in carrying out a CAA we conclude: i) REACH registration dossiers provide a comprehensive source of hazard information for an alternative assessment. It is important to consider that the presented data is subject to changes and its quality is variable. ii) Correct identification of the chemicals is crucial to retrieve the right data. This can be challenging for mixtures, reaction products or nanomaterials or when only trade names are available. iii) The quality of the data and the practice on how to fill data gaps can have a huge impact on the results and conclusions. iv) Current assessment criteria have mainly been developed for organic chemicals and create challenges when applied to inorganic solids, including nanomaterials. It is therefore crucial to analyse and report uncertainties for each decision making step.

## Highlights

• Chemical alternatives assessment is a useful tool to find safer flame retardants.
• Mainly bioaccumulation and persistence contribute to high hazard scores.
• MWCNTs can improve the performance of halogen-free flame retardants.
• Repeated inhalation toxicity is the major concern for MWCNT.
• Data availability and quality can have a huge impact on the assessment outcome.

## 1 Introduction

Flame retardants (FRs) are a diverse group of chemicals used as additives in a wide range of products to inhibit, suppress, or delay ignition and to prevent the spread of fire. They can act in a range of different ways, from interfering with fire's ability to consume oxygen, to forming a barrier or to acting as chemical coolants (Morgan and Wilkie, 2006). The increasing use of thermoplastics and thermosetting polymers for applications in buildings, transportation, electrical electronics and engineering together with increasing demands for fire safety have resulted in the research into a variety of FRs over the last decades (Visakh and Arao, 2015).

FRs are divided into reactive (chemically bound into the materials) and additive (integrated into the materials by physical mixing only) (Beard, 2013). They are usually classified based on their “active” chemical element – the main groups being products based on halogens (mostly bromine, but also chlorine), phosphorus, nitrogen, minerals such as aluminium and magnesium and to a lesser extend boron, zinc and carbon. Recently, also nanomaterials (NMs) such as nanoclay and multi-walled carbon nanotubes (MWCNTs) have been used as FRs or synergists (Beyer, 2003, Kashiwagi et al., 2005a).

Halogenated FRs (HFRs) have been the most commonly used compounds in many different applications as they can be added to plastics without altering the properties, while preserving the total colourability (commonly up to 5% in polyurethane foams in furniture and up to 20% in plastic of some electronic housings) (Shaw et al., 2010, American Chemical Council, 2016). They are also highly effective for reducing heat release rates of commodity polymers.

Bromine interacts with the fire cycle in the gas phase to stop the chemical chain reaction that leads to flame formation and a self-sustaining fire. In essence, brominated FRs either prevent a fire from starting in the first place, or significantly slow it down. The future use of some brominated FRs (BFR) is however becoming highly questionable as they have been identified as global contaminants and are associated with adverse health effects including potential reproductive, developmental, and neurological effects (Birnbaum and Staskal, 2004, ARCADIS, 2011). The most extensively used BFRs have been tetra-bromobisphenol A (TBBPA), hexabromocyclododecane (HBCD) and three commercial mixtures of polybrominated diphenyl ethers (PBDEs): decabromo-diphenylether (deca-BDE), octabromo-dipheylether (octa-BDE) and pentabromo-diphenylether (penta-BDEs) (Shaw et al., 2010). PBDEs and polybrominated biphenyls (PBBs) have been restricted in the EU since 2004 (European Parliament and Council, 2011) and the production of penta- and octa-BDE has been phased out in the US since 2004 (US-EPA, 2008). Penta-BDE and octa-BDE have been identified as persistent organic pollutants (POP) (SFT, 2009), and Penta-BDE is already listed as a hazardous substance under the Water Framework Directive (WFD), while octa-BDE and deca-BDE are listed among the substances to be monitored (European Parliament and Council, 2000). Deca-BDE and HBCD were included by the European Chemicals Agency (ECHA) in the list of candidates for Authorisation under REACH Article 57d as substances of very high concern (SVHC) based on PBT (persistent, bioaccumulating, toxic) properties.^1^ TBBPA is registered under REACH and is currently not subject to any REACH restriction processes. Tris(2-chloroethyl) phosphate (TCEP) is a representative of the chlorinated FRs and has been included in Annex XIV of REACH (“Authorisation List”) because of concerns over its reproductive toxicity (Cat 1B).

Humans may be exposed to HFRs entering the environment through multiple pathways, such as emissions during manufacturing, from products in use, leaching from landfills, combustion or recycling at the end of the products' life. Due to their persistence HFRs can travel long distances and bioaccumulate; some were even found in human breast milk (Shaw and Kannan, 2009, Fromme et al., 2016). Children's exposure to HFRs through indoor air dust has also been indicated in the literature (Mizouchi et al., 2015). Furthermore, HFRs are of concern because of their propensity to produce toxic fumes and acids (hydrobromic and hydrochloric acids, organo-irritants, dioxins/furans [PCDD/F]) during fire (Stec and Hull, 2010).

**Fig. 1:**
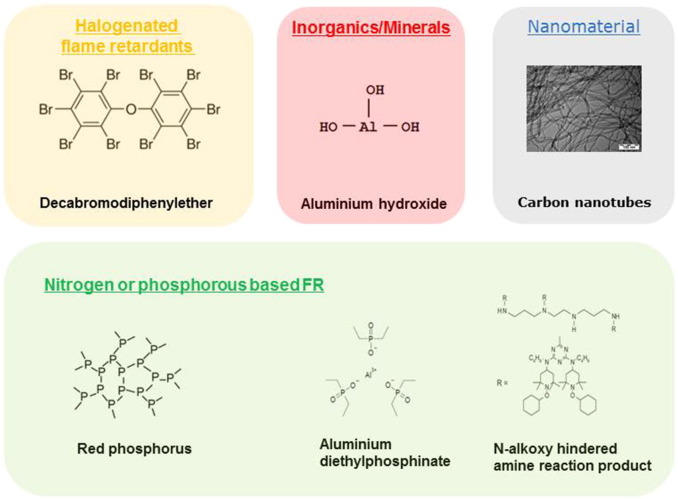
Overview of the classes of flame retardants assessed in this study and the chemical structure.

These concerns have initiated a search for alternative flame-retardant additives with comparable FR performance but with less adverse effects. A variety of halogen-free flame retardants (HFFRs) are available on the market, including organic (phosphorus and nitrogen based chemicals) and inorganic (boron and metals) (see Fig. 1). Some of them have been assessed as alternatives for FRs of concern (KemI, 2005, Danish EPA, 2006, Illinois Environmental Protection Agency, 2007, SFT, 2009, US-EPA, 2014a, US-EPA, 2014b, ENFIRO, 2016). Some FR producers have formed an industry group, PINFA (Phosphorus, Inorganic and Nitrogen Flame Retardants Association) (PINFA, 2016), with the goal to develop and promote more environmentally compatible products with focus on halogen free FRs (HFFRs). To be an effective HFFR, high loadings may be required, which could have an impact on the mechanical properties of the materials and products. Also, for some products the manufacturing process using HFFR may become difficult and energy-intensive.

The class of phosphorus-based FRs covers a wide range of inorganic and organic compounds: red phosphorus, phosphate esters, phosphonates and phosphinates (American Chemical Council, 2016, PINFA, 2016). In the presence of a heat source, phosphorus FRs release phosphoric acid which interrupts the combustion process by promoting the char formation due to incomplete combustion. Phosphorus FRs, which include organic and inorganic phosphates, phosphonates, and phosphinates as well as red phosphorus, are used in a broad range of applications (plastics, textiles, polyurethane foams, coatings and rubber), and have a good fire safety performance (PINFA, 2016).

Nitrogen containing FRs are mainly based on pure melamine, melamine derivatives or homologues. They are believed to act by several mechanisms (American Chemical Council, 2016, PINFA, 2016): at high temperatures, they enable the formation of cross-linked structures which promote char formation. The release of inert nitrogen gases inhibits the chain reaction by diluting combustible gases. Synergistic effects can be achieved by combining phosphorus and nitrogen, where nitrogen enhances the attachment of phosphorus to the fire-protected polymer. Nitrogen FRs are used in insulation, furniture foams and electronics.

The most common inorganic FRs are the hydroxides of aluminium and magnesium with aluminium trihydroxide (ATH) being by far the most widely used FR on a tonnage basis (American Chemical Council, 2016, PINFA, 2016). It is inexpensive, but usually requires higher loadings in polymers of up to > 60% and decomposes at around 200 °C. Magnesium hydroxide (MDH) is stable up to temperatures of around 300 °C and can thus be used in polymers which have higher processing temperatures. Antimony trioxide is often used as part of a FR system in combination with bromine, phosphorus or nitrogen FRs. Inorganic FRs are used in some plastics, paints, adhesives, rubber, textile back coatings, wires and cables. Their FR mechanism is based on the release of water which cools and dilutes the flame zone.

**Fig. 2:**
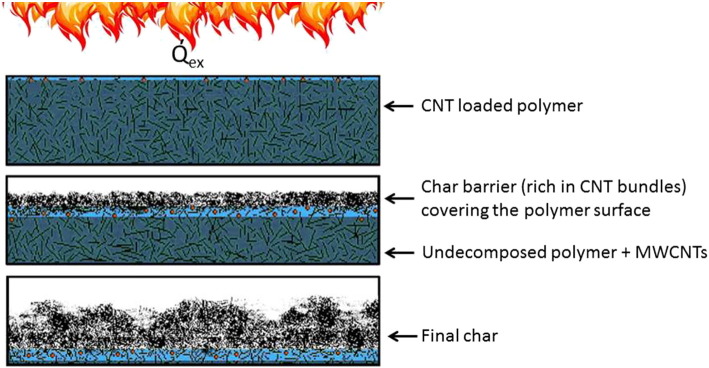
MWCNTs as fire protection promoters (adapted from (Kashiwagi et al., 2005b)).

Nanomaterials such as carbon nanotubes (CNTs) and nanoclays have been demonstrated to act as very effective/synergistic co-additive in some FR applications (Beyer, 2003, Kashiwagi et al., 2005a). When properly dispersed in polymer matrices they enable a reduction of the loading of other FRs and improve their fire performance (see Fig. 2) which is particularly interesting for HFFR. Polymer nanocomposites are also attractive because they have the potential to introduce true multifunctionality by improving other properties such as mechanical properties, conductivity and electromagnetic shielding (Morgan and Wilkie, 2006).

The main FR mechanism of CNTs during thermal decomposition is that they promote the formation of a char layer covering the polymer surface, acting as an insulating barrier. This barrier limits heat transfer and diffusion of oxygen into the material and reduces the escape of volatile, combustible degradation products to the flame (Kashiwagi et al., 2005b, Kashiwagi, 2007). In addition CNTs can increase the strength of the material, make it more fatigue-resistant and improve its electrical properties. CNTs act synergistically to other FRs and have for example been suggested for application in textiles or polymer composites (NANOCYL S.A., 2012, Nanotechproject, 2012, De Volder et al., 2013, US-EPA, 2013).

The synergistic effects of MWCNT on both material properties and fire safety performance of a set of FRs as well as their impact on human health and environment were investigated in the frame of the FP7 project DEROCA (Development of safer and more Eco-friendly flame Retardant materials based on CNT cO-additives for Commodity Applications; grant agreement n° 308391) (NANOCYL S.A., 2012).

The current paper focuses on the human health hazard assessment of selected FRs while considering also environmental fate and ecotoxicity. We present the results of the chemical alternatives assessment and discuss the challenges encountered and the uncertainties identified. This study delivers useful results and conclusions that can be used as part of a comprehensive alternatives assessment in the search for suitable alternatives to traditional HFRs of concern.

## 2 Process and methods

Chemical alternatives assessment (CAA) is a methodology for identifying, comparing and selecting safer alternatives to chemicals of concern (Lavoie et al., 2010, Whittaker and Heine, 2013, NAS, 2014, Geiser et al., 2015). Multiple methods and paradigms for CAA have been proposed and are in use by different organisations to address the challenge of substituting chemicals identified as having a negative impact on human health and/or the environment (CPSI, 2016; SubsPort, 2016). Most CAAs are based on a comparative chemical hazard assessment (CHA), although a comprehensive CAA can be much broader and include other factors such as costs, availability, performance, and social and environmental life cycle attributes (Whittaker and Heine, 2013, NAS, 2014, Geiser et al., 2015, Tickner et al., 2015). CHA methods address mostly human and environmental hazard endpoints and environmental fate. In CHA the endpoints are evaluated based on criteria that allow the use of measured or predicted data (US-EPA, 2011, CPA, 2013). CHA may also be used proactively in the development of new materials or products to eliminate inherent hazards in the design phase. (Whittaker and Heine, 2013, Hjorth et al., 2017). The benefit of identifying chemicals with lower hazards is that hazard is inherent, whereas exposure and thus risk can vary significantly as they depend on the application and exposure situation.

For our CAA of FRs we selected methods whose paradigms are fully transparent and which are publicly available, i.e. the ECHA's Chemical Safety Assessment (CSA) protocols (ECHA, 2008, ECHA, 2011), the Design for Environment (DfE) program of U.S.-EPA (US-EPA, 2011) and the chemical screening method Clean Production Action's (CPA) GreenScreen® (Clean Production Action, 2013). These programmes provide hazard data and/or scores for most of the substances of interest within the project allowing to rank alternatives with regard to desirability. Other methods as reported in (Whittaker and Heine, 2013, NAS, 2014) were explored but finally not applied as they were either not publicly available or considered to be too complex or not advanced enough to be easily applicable.

The U.S. EPA's DfE Program (US-EPA, 2016) characterises chemical hazards based on a wide range of human health and environmental information based on defined DfE criteria (US-EPA, 2011). Both experimental and modelled data are used in assigning a hazard designation as (Very) High, Moderate or (Very) Low concern (VH, H, M, L, VL). Coloured/bold letters imply that the designation is assigned based on empirical data (test results). Black italics letters are assigned to endpoints when values from predictive values or expert judgement are used.

The GreenScreen® for Safer Chemicals (version 1.2) was developed by the American NGO (non-governmental organisation) CPA. It is a chemical screening method using a benchmarking system to rank the relative hazard level of chemicals (CPA, 2013). Its structure builds on U.S.-EPA's DfE CAA programme and applies the same threshold values, although classification by GHS (globally harmonised system of classification and labelling of chemicals), IARC (International Agency for Research on Cancer), and EU-SVHC (Substances of very high concern) are also considered. In addition to DfE it assesses also endpoints like “Endocrine Activity”, “Immune System” and “Physical Hazards” (“Reactivity” and “Flammability”). Some GreenScreen® evaluations also consider the hazards of breakdown products in the overall assessment (Rossi and Heine, 2007). The result of each hazard endpoint is evaluated against criteria and finally scored to one of the following GreenScreen® Benchmarks: 1: Avoid – Chemical of high concern, 2: Use but search for safer substitutes, 3: Use, but still opportunity for improvement, 4: Prefer-Safer chemical.

We derived hazard designation and hazard scores by following the suggested methods and criteria. Contrary to the DfE program we did not endeavour to fill data gaps by the “analogue approach” to structurally related compounds (OECD, 2014). The selected methods do not directly address life cycle impacts, but the collected data and/or CHA results can feed into such assessments. Comparative life cycle assessments (LCA) of existing and alternative products have been carried out by other project partners (NANOCYL S.A., 2012) and elsewhere (ENFIRO, 2016, Gilbertson and Ng, 2016).

For the preparation of the CHA, we performed a comprehensive review of current literature and databases. This included relevant physico-chemical, fate and hazard data found in REACH registration dossiers (ECHA Registered substances, 2016), from the CAAs by U.S.-EPA (US-EPA, 2013, US-EPA, 2014a, US-EPA, 2014b, US-EPA, 2015b, US-EPA, 2015a) or other institutions (KemI, 2005, Danish EPA, 2006, Illinois Environmental Protection Agency, 2007, SFT, 2009) as well as in other available risk assessments of FRs (ARCADIS, 2011). For the hazard assessment we focused on biological assays adequate to meet the REACH information requirements, i.e. priority is given to results from studies following OECD test guidelines and carried out according to good laboratory practice or of comparable reliability (i.e. Klimisch code 1 or 2 (Klimisch et al., 1997)). Our conclusions are based on data included in the database at the time the search was performed. We recognise that the consulted databases (e.g. REACH registration dossiers) are living documents which are regularly updated and new evidence can potentially change the results and conclusions. Details on human health and environmental information for MWCNT as well as scoring criteria are presented in the supplementary material.

Within the DEROCA project different HFFR materials were tested which were selected by all project partners based on technical specifications relevant for the product type, fire performance (smoke, heat flux, toxicity) and hazardous properties. In the original study of the project, we evaluated and compared 4 HFR (decabromodiphenyl ether, decabromodiphenyl ethane, Hexabromocyclododecane, tris(2-chloroethyl) phosphate) and 7 HFFR (aluminium diethylphosphinate, ATH, ammoniumpolyphosphate, magnesium hydroxide, red phosphorus; MWCNT, *N*-alkoxy-hindered amine). In this article we focus on those substances which we found most interesting as either there was no CAA available or because of the challenges in evaluating the data and assigning hazard scores: Multi-walled carbon nanotubes, aluminium diethylphosphinate, aluminium trihydroxide, *N*-alkoxy hindered amines and red phosphorus.

A human health risk assessment of each FR was carried out by following the ECHA guidance for Chemical Safety Assessment under REACH (ECHA, 2008, ECHA, 2009). In addition to the Derived No Effect Levels (DNELs) provided in the REACH registration dossiers we made our own estimations for chronic inhalation exposure and compared the values. DNELs are derived from dose descriptors such as No-Observed Adverse Effect Levels (NOAELs) from repeated dose toxicity studies by applying assessment factors (AFs) to account for differences between and among species, duration and different exposure routes. DNELs were used to rank the FRs.

## 3 Results

### 3.1 Hazard assessments of FRs

The most relevant endpoints for the hazard assessment of the selected FRs, their DNELs and classification and labellling proposals according to the criteria of Regulation (EC) No 1272/2008 on Classification, Labelling and Packaging of Substances and Mixtures (CLP) (European Parliament and Council, 2008) are described below. The results are summarised in Table 1. Hazard designation according to DfE criteria (US-EPA, 2011) (see Section 2) are provided for each endpoint in brackets in the text and summarised in Table 2. The results of the GreenScreen® assessment are provided in Table 3. We adopted the hazard designations as assigned by DfE and the GreenScreen® scores, when available and not in contradiction to the data that was available to us. In case they did not conform, we describe and discuss the differences.

#### 3.1.1 Decabromodiphenylether (deca-BDE, BDE-209)

Deca-DBE (Bis(pentabromophenyl)ether, CAS no 1163-19-5) is the most widely used form of the PBDEs. It has become reference substance for several alternative assessments (KemI, 2005, Danish EPA, 2006, Illinois Environmental Protection Agency, 2007, Rossi and Heine, 2007, US-EPA, 2013, US-EPA, 2014a). An EU risk assessment was finalised in 2002 (ECB, 2002) and deca-DBE is registered under REACH (ECHA Registered substances, 2016). In the EU Deca-BDE has been proposed as substance of very high concern by meeting the criteria of a PBT/vPvB (very persistent, very bioaccumulating) substance (ECHA, 2012) and is currently being considered for listing on the Stockholm Convention for persistent organic pollutants (POPs) (Stockholm Convention, 2016). Deca-BDE like other higher brominated congeners have lower bioaccumulation potential, water solubility and volatility than lower brominated congeners, however, their breakdown products are likely to be more bioavailable in the environment and food web (Huang et al., 2010). Some metabolites of deca-BDE are known to produce estrogenic effects and deca-BDE has been listed as potential endocrine disrupter (Cat 2) on the EU Priority List of Suspected Endocrine Disrupters.^2^ The main endpoint of concern for human health is developmental toxicity (Costa and Giordano, 2011, US-EPA, 2014a). The REACH dossier and most notifications propose no classification for human and environmental hazard according to CLP Regulation (ECHA C&L inventory, 2016, ECHA Registered substances, 2016).

Upon oral administration deca-BDE has shown limited absorption (10–25%) and rapid excretion through the faeces (Hardy et al., 2009, Costa and Giordano, 2011). Monitoring studies in humans demonstrate that deca-BDE can be absorbed, distributed to mammary tissue and secreted in human breast milk during lactation (US-EPA, 2013). No information upon inhalation and dermal absorption is available but absorption is expected to be rather low. Due to contamination in dust and other particles, dermal and oral exposure seem to be the primary exposure routes for consumers, which could particularly concern toddlers (La Guardia and Hale, 2015, Mizouchi et al., 2015, Sahlström et al., 2015). Deca-BDE exhibited low (**L**) acute toxicity via all routes of exposure (US-EPA, 2013). Subchronic and chronic studies suggest a moderate (**M**) concern, based on adverse liver and thyroid effects observed in rats. The NOAELs of chronic studies were determined to be 1120 mg/kg bw which allow a sufficiently high margin of safety for worker and consumer exposure (ECB, 2002). Deca-BDE does not appear to be a skin sensitiser or irritant based on observations in animals and humans; neither does it appear to be an eye irritant (**L** for all these endpoints) (US-EPA, 2013). No data on respiratory sensitisation is available.

The absence of gene mutations in bacterial and mammalian cells and lack of chromosomal aberrations in Chinese hamster ovary (CHO) cells in vitro suggest that deca-BDE is not genotoxic (**L**). The carcinogenic potential was considered moderate (**M**) by U.S.-EPA based on an NTP (National Toxicology Program) study (NTP, 1986) demonstrating evidence of carcinogenicity (liver and thyroid) in more than one species, sex and site (US-EPA, 2013). IARC concluded that deca-BDE is not classifiable as a human carcinogen (Group3) based on limited evidence in animals (IARC 1998) and the EU-RAR (Risk Assessment Report prepared under the Existing Substances Regulation No 793/93) concluded that the observed effects did not warrant a classification for carcinogenicity (ECB, 2002). Deca-BDE showed no reproductive effects at doses exceeding 1000 mg/kg-day (**L**) whereas a number of rodent developmental neurotoxicity studies showed reduced thyroid hormone levels and abnormal behaviour leading to a concern especially about neurodevelopmental toxicity (**H**) (US-EPA, 2013). There are however several studies, including guideline studies, which provide no evidence of adverse effects on neurodevelopment (Costa and Giordano, 2011) and also the EU-RAR concluded that no concern for adverse effects on development may be assumed (ECB, 2002). Despite these discrepancies we kept the hazard designations suggested by DfE. Concerning adult neurotoxicity, there is no reported evidence to support a hazard potential for this compound or analogous highly brominated compounds (US-EPA, 2014a) (*L*).

The acute and chronic aquatic toxicity is considered low (*L*) based on studies carried out with fish, daphnia and algae (ECB, 2002, US-EPA, 2014a).

Deca-BDE is not expected to degrade rapidly under aerobic conditions and considering that its primary degradation half-lives in sediment and soil significantly exceed 180 days the substance is classified very persistent (**VH**). The potential for bioaccumulation is expected to be high (*H*) based on monitoring data indicating that deca-BDE accumulates in higher trophic level organisms.

A long-term inhalation DNEL of 6 mg/m^3^ derived by applying an AF of 25 has been suggested in the REACH dossier. As no inhalation study is available, the DNEL was extrapolated from the oral route. No details are provided, but it can be assumed that it was derived by considering an absorption rate of < 10% for the oral route and 100% via inhalation from a chronic NOAEL of 1120 mg/kg/day as was done in the EU RAR (ECB, 2002).

#### 3.1.2 Multi-walled carbon nanotubes (MWCNTs)

Multi-walled carbon nanotubes (MWCNT) consist of multiple rolled layers (concentric tubes) of graphene. Depending on the synthesis and purification methods (Prasek et al., 2011) they may differ in their form (length, diameter), rigidity and other physico-chemical properties (metal content, e.g. Fe, Co, Ni, aggregation, agglomeration, surface chemistry and functionalisation) which may all have an impact on their toxicological profile (Donaldson et al., 2010, Braakhuis et al., 2014, Poulsen et al., 2016).

In our review we focus on two types of MWCNTs that were registered under REACH with list number 936-414-1 (MWCNT Dossier, 2016). Most of the studies in this dossier were carried out with either Nanocyl NC7000™ or Baytubes® 150CP, which are short, tangled MWCNTs obtained by catalytic chemical vapour deposition and which are not surface modified. Based on these data and complementary information, we carried out an analysis of MWCNT properties and (eco)toxicological effects following the pattern of an U.S.-EPA DfE CAA and GreenScreen® Assessment. These assessments are presented in detail in the supplementary material; the results are summarised below. NC7000™ have an average diameter of 9.5 nm and an average length of 1.5 μm (Wohlleben et al., 2013, Nanocyl, 2016); Baytubes® 150CP have a diameter of 10 nm and tube lengths in the range of 200–300 nm with some tubes resulting in higher length medians of 1000 nm (all sizes determined by Transmission Electron Microscope) (Pauluhn, 2010a). The BET (Brunauer–Emmett–Teller theory) surface area analysis gives a value of 250–300 m^2^/g. The carbon purity of both types is higher than 90%, sometimes reported to be 99% (Thermogravimetric analysis) while the metal oxide concentration is below 10% (Inductively Coupled Plasma Mass Spectrometry). The most important (metal) impurities are Al, Fe and Co. MWCNT are not soluble in water and are not readily biodegradable (Rasmussen et al., 2014, OECD, 2015).

Based on the similarity of physico-chemical properties of the two MWCNT types an implicit read across between results from both types of MWCNTs was applied in the registration dossier (MWCNT Dossier, 2016). This approach is adopted, taking into account that nanoforms of the same chemical composition may induce different toxicological effects and therefore a scientific justification for read across is needed endpoint by endpoint (ECHA et al., 2016). Any conclusions drawn in this assessment are valid only for the described MWCNT types and may not be relevant for other CNTs with other dimensions and properties (Gilbertson and Ng, 2016). Studies with other types of MWCNTs were taken into consideration to support or discuss the conclusions obtained with the two MWCNT types.

No hazard classification according to CLP Regulation (European Parliament and Council, 2008) has been proposed. MWCNTs list No 936-141-1 together with the other REACH registered MWCNTs are included in the Community Rolling Action Plan for substance evaluation under REACH^3^ for the following reasons: suspected C (Carcinogen), wide dispersive use, exposure of environment, consumer use, cumulative exposure, exposure of workers, environmental fate and ecotoxicity.

Following oral administration of ^14^C labelled MWCNTs no translocation from the GI-tract was observed into systemic circulation or any of the organs investigated (spleen, liver and lung) (Jacobsen et al., 2013). A study report (MWCNT Dossier, 2016) suggested oral absorption, based on observed granulomatous changes in liver and MWCNT in urine, however without findings of MWCNTs in liver and kidneys. MWCNTs showed no permeability in a validated Caco2-cell assay suggesting that they do not cross intestinal epithelium and do not enter the systemic blood circulations (MWCNT Dossier, 2016).

No adverse effects (toxicity, sensitisation) and no evidence for systemic availability of MWCNTs after dermal exposure was shown in vivo and in vitro (MWCNT Dossier, 2016). Absence of cytotoxicity (Vankoningsloo et al., 2010) in a skin cellular model suggests that MWCNTs are not able to enter the epidermis through the corn barrier. After both single and repeated intravenous (IV) injections to rats, most of MWCNTs were observed in liver and lung, with a few percent present in spleen, kidneys, heart and testes (Jacobsen et al., 2013). MWCNTs were biopersistent in liver and lung beyond 3 months after administration. Following single and repeated pulmonary exposure (inhalation and intratracheal instillation) MWCNTs were observed to remain within the lungs for several months (Muller et al., 2005, Ellinger-Ziegelbauer and Pauluhn, 2009, Pauluhn, 2010a). At overload conditions clearance from the lung was markedly delayed. Following sub-chronic exposure translocation into lung associated lymph nodes (LALNs) occurred in a time dependent manner (Pauluhn, 2010a). Lung burdens and kinetics of MWCNTs have shown to be highly dependent on the disaggregation of aerosolized MWCNT, a property which is influenced by the technical processes to formulate MWCNTs (Pauluhn and Rosenbruch, 2015).

Following single exposure MWCNT showed low (L) oral (LD_50_ ≥ 5000 mg/kg bw) and dermal (LD_50_ > 2000 mg/kg bw) toxicity (MWCNT Dossier, 2016). The acute LC_50_ for rat inhalation exposure is > 241 mg/m^3^, which was the maximum technically attainable concentration (Pauluhn, 2010a). The acute No-Observed Adverse Effect Concentration (NOAEC) is 11 mg/m^3^ air. At the highest tested concentration no deaths and only transient clinical signs (irregular and laboured breathing patterns and reduced body weights) were observed. This concentration is lower than the threshold LC_50_ value (< 500 mg/m^3^) for very high hazard, however as the effects observed at this concentration were mild, a hazard assignation of very high does not seem to be appropriate and a score of *Moderate* (*M*) (usually for values 1–5 mg/L/d) is proposed, as it seems very unlikely that concentrations inducing severe effects can be generated.

MWCNTs were not irritating to skin and eye and not sensitising to skin ((V)L) (Kishore et al., 2009, Ema et al., 2011, MWCNT Dossier, 2016). MWCNT seem to have (*L*) potential to induce allergic reactions in the respiratory tract (Safe Work Australia, 2012).

Repeated sub-chronic exposure of rat to MWCNTs induced sustainable lung inflammation and lead to translocation of MWCNTs to lung associated lymph nodes (Ma-Hock et al., 2009, Pauluhn, 2010a); no systemic toxicity was observed. Due to the low NOAEC/LOAEC (Lowest Observable Adverse Effect Concentration) of 0.1 mg/m^3^, MWCNT are of considered of *high* hazard (*H*) for repeated inhalation exposure (threshold for H is a NOAEC ≤ 20 mg/m^3^). No oral or dermal study is available, as these are not considered relevant exposure route; in any case hazards are expected to be low (*L*) due to lack of translocation/absorption via these routes.

The potential for genotoxicity is considered low (*L*). Out of 29 in vitro studies performed with NC7000 and Baytubes 80% were negative (for details see Supplementary material). From the available 8 in vivo tests with NC7000 one showed a significant increase of nucleated AT-II-cells (ex vivo) in bronchoalveolar lavage following intratracheal instillation of 9.1 mg/kg, which was not dose dependent (Muller et al., 2008). Observed positive responses were low and may represent secondary genotoxicity (Nanogenotox, 2013) following oxidative stress at high concentrations.

A concern regarding carbon nanotubes, due to their fibre like structure and high aspect ratio, is that they potentially induce mesotheliomas like asbestos fibres. Results from intraperitoneal injection of short, tangled MWCNT (Nanocyl NC 7000) indicated that they were not carcinogenic (Muller et al., 2009, Xu et al., 2012) in rat, whereas long MWCNT (MWNT-7) following intraperitoneal and intrascrotal injection induced mesotheliomas in mice (Takagi et al., 2008, Sakamoto et al., 2009, Takagi et al., 2012). Important factors for the formation of mesothelial carcinomas are the solubility/durability, length, diameter and shape that influence the clearance from lung, the entry into and clearance from mesothelium. The longer and more rigid/needle-like a MWCNT fibre is the higher the risk, the shorter and the more bent, curved or waved the lower the toxic and carcinogenic potency seems to be (Donaldson et al., 2010, Rittinghausen et al., 2014). Also chemical groups on the surface are suggested to contribute to the development of fibrosis which can result in carcinogenesis (Donaldson et al., 2010). Based on the short length and the propensity to tangle low (*L*) hazard assignation is suggested for NC7000 and Baytubes®. In 2014, IARC classified MWCNT-7 (Diameter: 74 ± 28 nm, length: 5.7 ± 3.7 μm) as possibly carcinogenic to humans (Group 2B) based on the evidence of observed mesothelioma following intraperitoneal (or intrascrotal) injection in rats. All other SWCNTs and MWCNTs were categorised in Group 3 as not classifiable as to their carcinogenicity to humans (Grosse et al., 2014).

MWCNT are estimated to be of low hazard (*L*) for reproductive and developmental effects due to expected low systemic availability and no indications for such effects from subchronic inhalation studies and prenatal developmental toxicity studies (MWCNT Dossier, 2016). Developmental effects were seen for other MWCNT types than those assessed in this study at concentrations causing maternal toxicity and when using non-physiological routes of exposure (IV injection, intraperitoneal injection, intratracheal instillation) (Hougaard et al., 2013, Ema et al., 2016). Chronic inflammation is considered to interfere with reproductive parameters.

A major concern for most FRs is their persistency and bioaccumulation. High persistency applies to all MWCNT types (*H*^*R*^) as they are recalcitrant (^*R*^) to biodegradation. Bioaccumulation is not likely due to the high molecular weight and diameter of MWCNTs (*L*), however, as some studies indicate uptake in plants and fish, further investigations into that issue are warranted (Petersen et al., 2011).

MWCNTs registered under REACH with list number 936-414-1 have shown low (L) acute and chronic toxicity to aquatic organisms at different trophic levels. No effects were observed up to the saturation limit (MWCNT Dossier, 2016). A recent CAA including MWCNTs concluded on a high acute and moderate chronic aquatic toxicity without specifying the MWCNT type (Gilbertson and Ng, 2016).

Our assessment shows that for most of the assessed hazard endpoints there is low concern. Based on a low NOAEC/LOAEC (0.1 mg/m^3^) the inhalation route is considered to be of highest concern, especially where exposure to free MWCNTs is possible, for example at the work place. A high designation was therefore suggested to be assigned for repeated dose inhalation toxicity in the DfE and GreenScreen® assessment, together with persistence (see Supplementary material and Table 2 and Table 3 below). Depending on how the criteria are applied to concerns over repeated inhalation toxicity a benchmark score of 2 (Use but search for safer substitutes) or 3 (Use, but still opportunity for improvement) can be assigned. Following a conservative approach we propose a score of 2 (Table 3).

The NOAEC/LOAEC of 0.1 mg/m^3^ from the subchronic inhalation (Pauluhn, 2010a) study serves as starting point to derive a DNEL for the risk characterisation. In the REACH dossier a DNEL of 50 μg/m^3^ is proposed by applying an AF of 2 taking into account differences in alveolar deposition, differences in ventilation, and the time-dependent particle accumulation accounting for the known species-specific differences in particle clearance half-times in rats and humans (Pauluhn, 2010b, MWCNT Dossier, 2016).

The US National Institute for Occupational Safety and Health (NIOSH) proposed a recommended exposure limit (REL) of 1 μg/m^3^ for carbon nanotubes based on the limit of quantification (NIOSH, 2013). The NIOSH value is in the same range as would be a DNEL derived from the NOAEC/LOAEC by applying default AFs (overall AF of 100) following the REACH guidance (Aschberger and Christensen, 2010, Aschberger et al., 2010). The DNELs are summarised in Table 1. No DNELs for the oral and dermal route were available.

#### 3.1.3 Aluminium diethylphosphinate (AlPi)

Aluminium diethylphosphinate (AlPi) is commercially available in products with trade names such as Exolit OP930, Exolit OP935 and Exolit OP1230 (Rakotomalala et al., 2010, Horrocks et al., 2014, US-EPA, 2015a, Clariant, 2016).

Currently, AlPi (named as Phosphinic acid, *P*,*P*-diethyl-, aluminium salt (3:1), CAS No 225789-38-8, list number 607-114-5) is under pre-registration process (ECHA pre-registered substances, 2016). Health and environmental hazards of AlPi have been recently assessed by U.S.-EPA DfE (US-EPA, 2015a). In many cases experimental studies regarding AlPi based substances are confidential and not directly accessible, but collected and retrievable from secondary sources.

Following oral exposure AlPi was only partially absorbed through gastrointestinal tract and mainly excreted in the faeces (US-EPA, 2015a), and not via urine as previously reported (Stuer-Lauridsen et al., 2007).

AlPi exhibits low acute toxicity (**L**) via the oral and dermal route of exposure (LD_50_ > 2000 mg/kg in rats) (US-EPA, 2014a). Low oral subchronic toxicity has been observed (28 day NOAEL > 1000 mg/kg-day in rats) (Stuer-Lauridsen et al., 2007) while chronic toxicity data are not located. The U.S.-EPA DfE assessment estimates for AlPi a moderate (*M*) repeated dose toxicity based on the comparison with analogous compounds.

The absence of induced genetic mutations and chromosomal aberrations in bacterial and mammalian tests suggests that AlPi is not genotoxic (**L**). The U.S.-EPA reports low concern for carcinogenicity (*L*) on the basis of expert judgement comparing AlPi to structurally similar metal salts (US-EPA, 2014a). No reproductive effects were observed up to 1000 mg/kg-day and low (**L**) (US-EPA, 2015a) hazard was assigned.

Although there is lack of experimental data, a moderate potential (*M*) is expected according to expert judgement for neurodevelopmental effects induced by the presence of phosphinate (US-EPA, 2014a). Notably, both U.S.-EPA and GreenScreen® (CPA, 2016a) estimate a moderate concern (*M*) for AlPi neurotoxicity by analogy to ATH and potential dissociation into ion (Al^+++^) constituents which are known to exert neurotoxic effects (Kumar and Gill, 2009). However, as recently discussed (Hendriks and Westerink, 2015), some in vitro and ex vivo studies suggest low neurotoxic potential for AlPi thus supporting the need of further investigations of neurotoxic endpoints.

According to observations from in vivo studies, AlPi appears not to be skin irritating (**VL**) and low potential hazard is considered for both skin sensitisation and eye irritation (**L**) (US-EPA, 2014a). No data is otherwise available for respiratory sensitisation.

A GreenScreen® assessment attributes to AlPi an overall benchmark score of 2 (Use but search for safer substitutes) based on moderate classification of some Group I and II human toxicity endpoints, such as developmental toxicity, repeated dose systemic and neurotoxic endpoints (Table 3). There are hazard data gaps for endocrine activity as well as single systemic toxicity, single neurotoxicity and respiratory sensitisation (CPA, 2016a).

Both acute and chronic aquatic toxicity of AlPi are moderate (**M**) based on experimental investigations performed with fish, algae and *Daphnia magna* (Waaijers et al., 2013, US-EPA, 2014a).

Concerning environmental fate, AlPi is estimated to be highly persistent (*H*^*R*^) because its metal ion is recalcitrant to biodegradation, whereas low risk (*L*) for bioaccumulation is expected (US-EPA, 2014a).

We calculated a DNEL of 58.3 mg/m^3^ following the REACH guidance (ECHA, 2010) starting from an oral NOAEL > 1000 mg/kg-day, as no inhalation study is available. This value is derived, assuming an absorption rate of 100% for both routes and applying an AF of 30 (5 for worker and 6 for duration extrapolation, the interspecies factor is already considered in the rat oral to human inhalation conversion). The proposed DNEL is comparable to a long-term worker inhalation DNEL of 63.2 mg/m^3^ with AF of 30 that we were able to retrieve from the REACH dossier of phosphinate Exolit OP 930 (Exolit OP930 Dossier, 2016).

#### 3.1.4 Aluminium trihydroxide

Aluminium trihydroxide (CAS No 21645-51-2) is the most widely used FR based on tonnage (European Flame Retardants Association, 2007). ATH is registered under REACH (ATH Dossier, 2016) reporting a production/import volume of 1000,000–10,000,000 tonnes per year. ATH does not meet the criteria for classification under CLP regulation.

ATH is estimated to be relatively non-bioavailable by any exposure route. The studies presented in the REACH dossier (ATH Dossier, 2016) show that the kinetics of aluminium uptake from the gastrointestinal tract is subject to significant inter-individual variability and depends on the aluminium species. The oral aluminium bioavailability from ATH in humans is reported to be low (< 0.1%) and to decrease with higher doses (Krewski et al., 2007). Following oral exposure, aluminium distributes throughout the organism and accumulates in bone, kidneys and brain. Different effects of concern with evidence of renal dysfunction, anaemia or neurobehavioural alterations have been reported after exposure to excessive doses of aluminium in humans (Krewski et al., 2007), however the concern was rated as low (**L**).

Based on data presented in the REACH dossier ATH has low potential for acute and chronic toxicity in mammals (oral LD_50_ > 2000 mg/kg bw in rats (gavage) and oral NOAEL > 302 mg/kg (diet)), while U.S.-EPA and GreenScreen® (CPA, 2014a) have estimated a moderate (*M*) concern for systemic toxicity (repeated dose) potential based on immunotoxic effects (reduction in primed cytotoxic T-cells) at low doses detected in human studies (LOAEL = 25 mg Al/kg-day). The impact of aluminium on the immune system is still controversial (Zhu et al., 2014).

Both U.S.-EPA (2014a) and GreenScreen® (CPA, 2014a) categorise ATH as moderate (**M**) neurotoxicant based on oral repeated dose studies showing impaired learning in a labyrinth maze test and also on ATH being listed on a developmental neurotoxicants screening list (Grandjean and Landrigan, 2006). Although a large number of animal and human studies support neurotoxicant effects of aluminium, the categorisation of ATH as neurotoxicant is still under debate. A major criticism to these studies is inconsistent experimental designs, protocols and information of chemical purity of these studies, and the uncertainty about any indication of neurological damage in the absence systemic toxicity.

There is general agreement that ATH is of no concern for mutagenicity (**L**) as it did not cause mutations in bacteria and chromosomal aberrations in vitro; low concern for carcinogenesis (*L*) is based on the absence of observations following exposure to analogous chemicals in humans and animals.

No reproductive adverse effects (**L**) have been described following ATH exposure. Some neurodevelopmental effects after exposure to aluminium lactate were presented in one study (LOAEL = 100 mg Al/kg bw/day) (ATH Dossier, 2016).

ATH is considered as not irritating to eye and skin (**VL**) although some proposals for classification for these endpoints exist. ATH is considered to have low potential (**L**) as skin sensitizer based on the mouse local lymph node assay. GreenScreen® (CPA, 2014a) scored ATH as very high respiratory sensitizer, based on its inclusion in the asthmagens list by the Association of Occupational and Environmental Clinics.^4^

ATH is recalcitrant to biodegradation (*H*^R^) but is not bio-available, owing to its extreme insolubility in water and small bioaccumulation factor (< 250) (*L*). Photolysis is not expected, as ATH does not absorb light at environmentally relevant wavelengths.

The REACH registration dossier does not identify any hazard for aquatic organisms based on acute and chronic aquatic toxicity studies in different fish species (i.e. LC_50_ of 22.4 mg/L aluminium sulphate; 44.8 mg/L aluminium chloride), while U.S.-EPA DfE estimated ATH to have moderate (*M*) acute and chronic aquatic toxicity based on moderate toxicity to fish reported for aluminium salt (mean fish 96-h LC_50_ = 11.0 mg/L, predicted result) (OPPT, 2010). One GreenScreen® assessment has scored ATH to have very high potential for chronic aquatic toxicity based on an experimental result in algae (NOAEC 0.004 mg Al/L) (CPA, 2014a) while in another acute and chronic ecotoxicity are rated as moderate (CPA, 2014c).

The DNEL suggested in the REACH dossier for long term inhalation of workers resulted 10.76 mg/m^3^, using an overall AF of 9 and LOAEC = 33.5 mg/m^3^ as dose descriptor starting point. This value is higher than a DNEL calculated by following the REACH guidance (ECHA, 2010). Following the REACH guidance the DNEL would be of 1.30 mg/m^3^ when using a LOAEC of ≥ 50 mg/m^3^ from a subchronic inhalation toxicity study in hamster (90 d, OECD Guideline 413) with aluminium powder (pyro) as starting point, converted to a human inhalation No Adverse Effect Concentration of 13 mg/m^3^ and applying an AF of 10.

The toxicological data available on ATH is limited; most of the hazard assessment data presented in the cited sources derives from aluminium analogues, using expert judgement. The toxicological categorisation of ATH is still under debate and different sources assign very different classification scores: The REACH registration dossier reports no hazard classification whereas GreenScreen® (2014) assigns ATH a Benchmark Score of 1 translated as Chemical of High Concern based on potential concern as immunotoxicant, neurotoxicant, respiratory sensitizer and toxic to aquatic organisms. Thus, in view of the disparity of information and based on the data provided from the different sources, in our consideration of ATH we include our own proposal based on the data presented in the REACH dossier and in the U.S.-EPA-DfE and conclude on a moderate inherent hazard (benchmark 2). This classification may change with the availability of new and more reliable data. Both scores are presented in Table 3.

#### 3.1.5 *N*-alkoxy hindered amine reaction products

*N*-alkoxy Hindered Amine Reaction Products that at the time of our assessment were sold with the trade name Flamestab NOR 116 (CAS No 191680-81-6), (molecular weight 2261 g/mol), refer to the molecular name of *1,3-Propanediamine, N,N*″*-1,2-ethanediylbis-, reaction products with cyclohexane and peroxidized N-butyl-2,2,6,6-tetramethyl-4-piperidinamine-2,4,6-trichlor-1,3,5-triazine reaction products* (Srinivasan, 2001, English et al., 2003). The substance registered under the same CAS No is only pre-registered under REACH, therefore no toxicological information from the registrant is currently available.

We based therefore our risk assessment on the data the producer provided in the REACH dossier for a fully registered *N*-alkoxy hindered amine with the trade name CGL 116, (EC No 425-020-0) (CGL-16 Dossier, 2016). CGL116 has also been used as name for Flamestab NOR 116 and is described as a reaction products of *N,N′-ethane-1,2-diylbis(1,3-propanediamine), cyclohexane, peroxidized 4-butylamino-2,2,6,6-tetramethylpiperidine and 2,4,6-trichloro-1,3,5-triazine*. CGL 116 is described by the producer as a Substance of Unknown or Variable composition (UVCB). Due to the uncertainty with regard to substance identity all hazard designations are presented in black italic letters.

For classification under CLP, the REACH registration dossier reports the data to be conclusive but not sufficient for classification of CGL 116 for health hazards.

The REACH dossier reports low (*L*) acute inhalation and dermal toxicity (LD_50_ > 5000 mg/kg in rats is and LD_50_ > 2000 mg/kg in rabbits, respectively). No relevant signs of toxicity associated with the repeated exposure (*L*) to CGL 116 at the tested levels were reported (CGL-16 Dossier, 2016). CGL 116 was not irritant to skin and eye, not sensitising and not genotoxic in three in vitro tests (all *L*). Based on the absence of genotoxic and other relevant effects following repeated exposure, the carcinogenic risk is considered low (*L*). A NOAEL from a reproductive/developmental toxicity screening study of 1000 mg/kg bw/d suggests low (*L*) concern for reproductive and developmental effects. On the contrary, the U.S.-EPA DfE assessment (US-EPA, 2014a) assigned high hazard levels to Flamestab NOR 116 due to its potential effects on liver, blood, gastrointestinal tract after repeated exposure as well as reproductive (*H*) and developmental effects (*H*); delayed skeletal maturation effect were detected at concentrations above 1200 mg/kg/day (= LOAEL). This human hazard assessment was mainly based on data from studies on hindered amines with chemical analogy to Flamestab and the same scores were assigned in a GreenScreen® assessment (CPA, 2014b). The U.S.-EPA report also considers moderate potential for carcinogenicity (*M*) due to the lack of data on this endpoint (US-EPA, 2014a). Taking these data into consideration, Flamestab NOR 116 would be estimated to have a high toxicity profile. However, we consider that these conclusions should be carefully considered, as the toxicological assessment is based on expert judgement omitting a clear justification for the dose descriptors values used to support this classification and using data mainly from analogues. The U.S.-EPA DfE assessment refers to the assessment of these endpoints in the TSCA dossier on hindered amines; Tinuvin 144 and Chimassorb 944 are the representative analogues for the category class of hindered amines. The reproduction and developmental toxicity effects described above are associated with the exposure of rats to Tinuvin 144. However, unlike Flamestab NOR 116 (MW = 2261), Tinuvin 144^5^ is a hindered amine with low molecular weight (685 g/mol), and therefore its toxicokinetics properties are expected to be different and potentially also its toxicological profile. Also, the U.S.-EPA report clearly indicates that due to the chemical structure (Molecular Weight > 1000) Flamestab NOR 116 is expected to have poor absorption (US-EPA, 2014a).

The REACH registration dossier for CGL 116 reported acute and chronic aquatic toxicity tests with LC_50_ values of > 100 mg/L and NOECs of ≥ 10 mg/L respectively, pointing to low (L) toxicity. CGL 116 is not readily biodegradable (*H* for persistence) and not bioaccumulating (*L*) in fish (CGL-16 Dossier, 2016).

To derive DNEL values for inhalation route in workers, the REACH dossier proposes to use the general exposure limit for inhalable dust (10 mg/m^3^/d). A calculated DNEL starting from a NOAEL ≥ 1000 mg/kg-day established in two rat oral studies (28 day oral and a > 5 week reproduction/developmental toxicity screening test) and applying an AF of 300 (10 interspecies, 5 worker, 6 duration) results in an inhalation DNEL of 11.6 mg/m^3^/day which is within a comparable range.

#### 3.1.6 Red phosphorus

Red phosphorus is an allotropic form of phosphorus which consists of phosphorus tetraedra connected in a polymeric chain-like structure (Mitchell and Burrows, 1990). It is produced by heating of white phosphorus, a highly reactive and toxic allotrope (ARCADIS, 2011, US-EPA, 2015a). Phosphorus allotropes can exhibit very different toxicity profiles.

Red phosphorus is registered under REACH with assigned list number 918-594-3 (Red Phosphorus Dossier, 2016). It is classified as flammable (Category 1, solids) and harmful to organisms in the aquatic environment (Category Chronic 3) according to CLP Regulation (ECHA C&L inventory, 2016).

The solubility and availability for absorption of red phosphorus was assessed as rather low in an in vitro model that simulates the dynamic conditions of the gastrointestinal tract; no data is available on distribution and excretion (Red Phosphorus Dossier, 2016). According to expert judgement, red phosphorus is expected not to be absorbed through the skin and poorly absorbed from the lung and the gastrointestinal tract (US-EPA, 2014a).

The REACH dossier for red phosphorous reports two studies establishing high lethal doses (LD) for oral exposure of rats (LD_50_ > 10,000 mg/kg and > 15,000 mg/kg) and the acute toxicity of red phosphorus is considered low (**L)** considering the reliable experimental data available (US-EPA, 2014a).

For repeated dose toxicity no registered data are located and a low (*L*) potential has been estimated by expert judgement in the U.S.-EPA DfE assessment (US-EPA, 2014a). Although some animal studies reported toxic effects after repeated exposure to smokes by pyrotechnic mixtures containing red phosphorus, this toxicity was not specifically attributable to any of the components of the mixture (US-EPA, 2014a).

Regarding genotoxicity, there are three in vitro studies available in the REACH dossier where red phosphorus was estimated as non-mutagenic and non clastogenic under the reported experimental conditions (Red Phosphorus Dossier, 2016). U.S-EPA assigned a moderate (*M*) genotoxicity hazard according to expert judgement as genotoxic effects cannot be ruled out (US-EPA, 2014a). Similarly a moderate potential hazard has been also assigned by GreenScreen® (CPA, 2016b).

Carcinogenic, reproductive and developmental effects of red phosphorus are considered of low hazard (*L*) based on expert evaluation in the absence of experimental data (US-EPA, 2014a, Red Phosphorus Dossier, 2016). Concerning neurotoxicity, only one animal study showed increased locomotor activity with incomplete recovery upon exposure to aerosols containing red phosphorus and butyl rubber and the overall potential is estimated to be low (*L*) (US-EPA, 2014a).

In the REACH dossier red phosphorus is considered as not irritating to eye and skin although a moderate hazard (M) is assigned by U.S.-EPA based on experimental studies showing corneal injuries and skin irritation upon exposure. As observed in vivo, red phosphorus is not a skin sensitizer (L); no data is available for respiratory sensitisation (US-EPA, 2014a).

Both the acute and chronic aquatic toxicity of red phosphorus are considered low (L) based on experimental data reporting effects at concentrations above the substance water solubility (US-EPA, 2014a, Red Phosphorus Dossier, 2016). Regarding environmental fate, red phosphorus is expected to be highly (H) persistent in the environment with a slow hydrolysis rate as reported in the REACH dossier. A low (*L*) bioaccumulation is estimated due to its very low water solubility and uptake across biological membranes (US-EPA, 2014a).

In the REACH dossier, a DNEL of 4 mg/m^3^ is proposed for long term exposure of workers with no documented AF. As no repeated dose toxicity study with red phosphorus for any of the exposure routes was found, we were not able to calculate a DNEL.

GreenScreen® assigned a benchmark score of 2 indicating a moderated concern related to the reactivity of red phosphorus (CPA, 2016b). A major drawback associated with the use of Red phosphorus use could be the formation of highly toxic phosphine (PH3) and phosphoric oxyacids that may occur through slow reaction with water and air and during combustion. Polymeric micro-encapsulation of red phosphorus can be used to prevent phosphine release and to further improve its effectiveness as a flame retardant (Laoutid et al., 2009).

### 3.2 Ranking of flame retardants based on DNELs

Health exposure limits such as the DNEL or occupational exposure levels (OEL) reflect the potency of a chemical hazard and may in principle allow establishing distinctions between substances with low and those with high thresholds. We therefore rank the assessed FRs based on the proposed DNELs for chronic inhalation exposure of worker with values in descending order. In addition, we present the classification proposals and endpoints of concern for each of the assessed FRs (Table 1). The DNELs are taken as proposed in the REACH dossier. Other reference values identified or calculated are reported in case they differ significantly (see MWCNT) and have an impact on the ranking. A ranking based on DNELs may in principle enable the identification of FRs of higher concern, as those with low DNEL require a higher level of exposure control (i.e. protection). However, we noticed that substances of concern are found among the potential substitutes and they would not be identified based on a low DNEL. In our original study in the project, we included more substances from both classes, HFRs and HFFRs, which supported this observation.

DNELs are usually based on the lowest dose descriptor and do not necessarily take into consideration the severity of effects. This means that carcinogenic or reprotoxic effects at high concentrations may lead to a higher DNEL than mild effects at low doses. In addition, concerns about chemicals are often a combination of hazard and fate properties (persistency, bioaccumulation), which are not reflected by the DNEL. To address this we have included the C&L proposals (according to CLP Regulation). Some substances have a harmonised classification, agreed upon by experts from EU competent authorities which is legally binding within the EU. Others have been self-classified by the person who is responsible for placing the chemical substance on the market. In some cases this may lead to a plethora of even contradicting proposals (see Table 1 where for each FR Hazard (H) phrases with > 10 notifications are presented). For a non-expert it becomes quite difficult to decide on which classification to apply when no harmonised classification is available.

For our comparison of DNELs we choose those for chronic inhalation exposure as they were available for all selected substances. In most cases these DNELs were not derived from inhalation studies but by route-to-route (R2R) extrapolation from oral studies. This is critical for those substances where the differences in toxicological effects are not (only) quantitative due to different absorption rates but of qualitative nature. Inhalation of insoluble inorganic (nano)particles or MWCNTs will (most probably) not lead to systemic absorption and distribution but rather induce local effects in the lung, which potentially may lead to systemic effects (e.g. cardiovascular diseases), but are different from those induced following oral absorption (Rennen et al., 2004, Saber et al., 2014).

The overall AFs used to derive a DNEL may also be subject to large variations. The REACH guidance proposes default values which however can be replaced by chemical specific adjustment (assessment) factors when appropriate (quantitative) chemical-specific data are available (WHO, 2005, ECHA, 2010). For MWCNTs, a DNEL of 50 μg/m^3^ (Pauluhn, 2010b) and a recommended exposure level (REL) (NIOSH, 2013) 1 μg/m^3^ were suggested. Both values were the lowest reference values from the assessed substances and the lower one is even is in the range of the limit of detection requiring strict exposure control (NIOSH, 2013).

### 3.3 Ranking based on Design for the Environment (DfE) criteria

The U.S.-EPA's Design for the Environment (DfE) Program aims to identify chemicals and processes with less human health and environmental impact by considering also economic effects and product performance (Lavoie et al., 2010). U.S.-EPA has carried out several “Alternative Assessments for Flame retardants of concern” published in a series of reports focusing on different substances or different applications (US-EPA, 2013, US-EPA, 2014a, US-EPA, 2014b, US-EPA, 2015b, US-EPA, 2015a).

We critically analysed the results of these alternatives assessments against other available information on the substances (see descriptions in Section 3.1); in most cases we adopted the results from the most recently available U.S.-EPA reports (see reference in column 2, Table 2). Only in case the data available to us resulted in very different scores (e.g. *N*-alkoxy hindered amines) or when no evaluation was available (MWCNT) we present our own proposal, obtained by following the same criteria (US-EPA, 2011). The assessment for MWCNTs is summarised in Section 3.1.2 and details supporting the decisions are presented in the supplementary material. The Screening Level Toxicity Hazard Summary for selected FRs based on the U.S.-EPA DfE Criteria is presented in Table 2. Based on the assigned hazard and the severity of effects we made an appraisal to rank the FRs from the highest to the lowest concern (Table 2).

Analysing assigned hazards for all FRs shows that FRs of concern are mainly characterised by their persistence, bioaccumulation and/or a CMR effect. In fact the potential for bioaccumulation may result in long-term (low dose) exposure, which is a concern as such, but in combination with (severe) effects it is even more essential to find safer alternatives.

All investigated non-halogenated alternatives in this assessment are persistent as they are inorganic and therefore recalcitrant to biodegradation. However, as they are not bioaccumulating, they are not considered to be PBTs and vBvPs and thus not as substances of concern.

Moderate to high hazards were assigned to some hazard endpoints of the FRs. The major challenge in the MWCNT assessment was to assign hazard for acute and repeated dose toxicity based on the available dose descriptors (see Section 3.1.2) from acute and sub-chronic inhalation studies (see Section 3.1.2). No deaths were observed at the maximum technically attainable concentration of 241 mg/m^3^ in the acute inhalation study (Ellinger-Ziegelbauer and Pauluhn, 2009). This value is lower than the lowest LC_50_ pointing to a score of VH (LC_50_ = ≤ 500 mg/m^3^); however as the real LC_50_ is not known and only transient clinical signs were observed at the highest concentration, such a high score does not seem to be appropriate. A score of *M* was selected to still consider the concern over inhalation exposure. Based on the low NOAEC/LOAEC from two 90 day repeated dose toxicity inhalation studies of 0.1 mg/m^3^ (Ma-Hock et al., 2009, Pauluhn, 2010a) assigning a hazard of H (high) has been suggested. This positions MWCNT more or less to a moderate risk among the assessed FRs.

Our hazard evaluation showed a very different picture for N-alkoxy hindered amines. U.S.-EPA identified data gaps for several endpoints and based its evaluation on an analogy approach to other hindered amines. We found information for these endpoints in the REACH dossier of hindered amines with the same CAS-No which did not confirm the severity of the described effects. Based on the U.S.-EPA evaluation *N*-alkoxy hindered amines would be considered as substances of very high concern and would thus not represent a reasonable alternative to HFRs. We have included *N*-alkoxy hindered amines twice in the table, once at the higher (concern) level based on the U.S.-EPA assessment and once at the lower end of the list as substance of low concern based on our assessment.

### 3.4 Ranking based on CPA's GreenScreen® criteria

The GreenScreen® for Safer Chemicals (version 1.2) by the Clean Production Action is a chemical screening method using a benchmarking system to rank the relative hazard level of chemicals and encourage progress toward safer alternatives (CPA, 2013).

Protocols to perform a GreenScreen® assessment are publicly available. Beside “Certified” GreenScreen® Assessments (by Licensed GreenScreen® Profiler), less than a hundred public domain GreenScreen® assessments (“NON-VERIFIED”) are currently available, for example by Interstate Chemicals Clearinghouse (IC2, 2016) and the Pharos Project (Pharos, 2016), which include also some FRs assessed in this case study. Such assessments may differ in the individual scores and the benchmark scores. For red phosphorus, ATH and AlPi certified GreenScreen® assessments were publicly available on the GreenScreen® website (CPA, 2014a, CPA, 2016a, CPA, 2016b) and for phosphorous FRs the Danish Environmental Protection Agency has recently published GreenScreen proposals (Danish EPA, 2016). To determine the MWCNT score, we carried out an assessment following the GreenScreen® criteria (CPA, 2013). The results of all assessments are presented in Table 3 and details are displayed in the supplementary material. FRs are presented according to increasing benchmark scores, i.e. from the highest to the lowest concern.

This ranking suggests the following substances as Chemicals of High Concern, which should be avoided (Benchmark score 1): Aluminium hydroxide got a Score of 1 in the certified Assessment because of high concern for respiratory sensitisation (CPA, 2014a), whereas in the non-verified assessment this was a data gap that did not contribute to the scoring (CPA, 2014c). In addition, the chronic ecotoxicity was assigned a score of very high in the certified assessment (in comparison to medium in the non-verified assessment).

For *N*-alkoxy hindered amines only a non-verified assessment could be found. The Benchmark score of 1 was based on human toxicity endpoints regarding reproductive toxicity and developmental toxicity, repeated dose systemic toxicity and very high aquatic toxicity (acute and chronic) along with very high persistence (CPA, 2014b). These scores are derived from the U.S.-EPA DfE evaluation and, as mentioned in Section 3.1.5, most of the endpoints of concern are not supported by data presented in the REACH dossier. Based on the latter data we prepared our own proposal, which lead to a score of 3 (Use, but still opportunity for improvement). Depending on how the effects following repeated MWCNT inhalation exposure would be scored (*H* or *M*), a Benchmark score of 2 or 3 would be assigned. Red phosphorus and aluminium diethylphosphinate reached a Benchmark score of 2 (Use but Search for Safer Substitutes), which was also suggested for deca-BDE. Notably, the breakdown products of deca-BDE, penta-BDE and octa-BDE have a score of 1 (avoid - chemical of concern), contributing to a Benchmark score of 1 for deca-BDE when also taking its breakdown products into consideration (Rossi and Heine, 2007). Besides deca-BDE no other GreenScreen® assessment was found that took breakdown products into consideration for the Benchmark score. The only substance we could find with a benchmark score of 4 (Prefer-Safer chemical) was water.

### 3.5 Consideration of life cycle stages and smoke toxicity

The consideration of life cycle impacts plays an important role in comparing alternatives in order to prevent shifting burdens between stages of the material's life cycle (NAS, 2014, ENFIRO, 2016). This is particularly important for functional substitutions where decisions are made based on data from heterogeneous solutions and not only on chemical properties (Tickner et al., 2015, Gilbertson and Ng, 2016, Hjorth et al., 2017). A life cycle risk assessment of FRs comprises occupational exposure during the production phase of FRs and the process of incorporation in the matrix, consumer exposure in dwellings under normal living conditions which can lead to inhalation of FRs released and accumulated in dwelling air and/or skin contact with substances migrated from products, such as furniture. During accidental fire, the properties of fire and the toxicity of combustion effluents have an important impact on the safety. At the end of the life cycle occupational exposure can potentially occur during recycling and/or disposal. Pollution and environmental releases can potentially occur at all stages of the life cycle, though this should be avoided.

As seen from the comparisons above, the major source of concerns arises from persistency and bioaccumulation as these properties facilitate a widespread exposure of humans and the environment. Substituting these substances by those with lower potential for bioaccumulation may in some cases lead to giving preference to substances with a higher toxicity profile. This would be the case for MWCNT where the risk could be shifted to the workplace where direct exposure to (high concentrations of) the pure chemical is most likely. However at this part of the life cycle exposure can be more effectively controlled (NIOSH, 2013). At later stages of the life cycle the exposure risk may be reduced due to embedding into matrices from which the NM based FRs are barely released (Wohlleben et al., 2015). Their low tendency to migrate from polymers (Šimon et al., 2008, Bott et al., 2014) and the low risk to be volatised seems to be one of the major benefits of solid inorganic compounds such as ATH or MWCNT. However at the stage of waste treatment, concerns for different materials may change. For HFR emissions in the use phase, through volatilisation, wearing or abrasion, cause the highest impact on human and freshwater exposure (Mizouchi et al., 2015). Although such emissions give only a small contribution to the overall impact over the complete life cycle, they represent the most important exposure route for humans. In contrast HFFRs may contribute more to the terrestrial and marine ecotoxicity (ENFIRO, 2016).

A comparison of the selected chemicals concerning their smoke toxicity was not carried out for the reason that fire effluents, including smoke and soot, are always toxic, irrespectively of the products burning. In any fire a few components affect the toxicity of combustion gases, with carbon monoxide always being present and, together with hydrogen cyanide, being predominant in most cases (Alarie, 2002). The generation of toxic combustion products is not only a material property but is strongly dependent on the amount of available combustible material, on the fire scenario (e.g. room size, temperature, ventilation) and on exposure time. However it was shown that HFFRs (ATH, AlPi, ammonium polyphosphate and magnesium hydroxide) had lower emissions of combustion products to air than HFRs (e.g. Tetrabromobisphenol A) (ENFIRO, 2016). Most HFRs produce smokes of high density and toxicity and can also contribute to dioxin formation due to improper treatment of waste of electrical and electronic equipment. The impact of HFRs in an accidental fire is in general more negative due to higher rate of smoke formation and higher terrestrial ecotoxicity score (ENFIRO, 2016). Halogen-free systems have demonstrated clear benefits: less and visible smoke, lower peak heat release rate, less toxic components in smoke.

Another potential health risk associated with FRs is the exposure of firefighters (or others) during clean-up after fires, as elevated rates of cancers related to dioxin exposure have been observed (Shaw et al., 2010). Following burning of MWCNT-containing polymers at temperatures below 600 °C, MWCNT could not be destroyed and remained in the residual ashes which may be aerolised and potentially lead to exposure (Chivas-Joly et al., 2014, Christou et al., 2016).

### 3.6 Addressing uncertainties in the chemical hazard assessment

Every decision in a risk assessment or chemical alternatives assessment is associated with variabilities and uncertainties which may have a significant impact on the results and their interpretation. A modern risk-assessment framework requires not only that the scientific evidence but also that the evaluation of the evidence and any judgments about the quality and relevance of the evidence to the risk assessors are thoroughly and clearly described (NAS, 2013, NAS, 2014, EFSA Scientific Committee, 2016). We have made an appraisal of potential uncertainties associated with the data collection and data evaluation in our case study and endeavoured to assess them semi-quantitatively (see Table 4) by describing them as low, moderate or high (L, M, H) according to the degree of their impact on the hazard estimation.

The verification of the identity of a chemical is the first step to enable the consultation of the appropriate hazard data for the assessment. Information can be found on manufacturer's websites, in the scientific literature and REACH registration dossiers, though the characterisation may sometimes not be detailed enough. A chemical can be identified by a name that is in accordance with the nomenclature systems of the International Union of Pure and Applied Chemistry (IUPAC) or the Chemical Abstracts Service (CAS), or a technical name. Different forms of the same chemical identity may exist, as for example NMs, which may exhibit different physico-chemical and/or biological effects. In the case of MWCNT, no CAS number and only a list number from the REACH registration was available. MWCNTs exist in different forms which influences their biological properties (Donaldson et al., 2006, Braakhuis et al., 2014). In our assessment we focused on two specific forms of MWCNT as covered by the list number to keep the variability as small as possible. In the case of reaction products such as *N*-alkoxy hindered amines (see Section 3.1.5) only the trade name was available to us. We consulted data that had been submitted for the same CAS number in the REACH registration dossier. Our conclusions based on this data were very different for several endpoints from the conclusions by U.S.-EPA for *N*-alkoxy hindered amines (US-EPA, 2014a). We therefore assume that data from studies with a substance of a different identity was probably consulted in that case.

A variety of FRs with the trade name Exolit OP exists with differences in the combination and dosage of their components (Clariant, 2016). When trying to find information on compounds of interest for us (Exolit OP930, OP935 and OP1230), we focused on their main constituent aluminium diethylphosphinate, a substance which is currently under pre-registration (no data has been submitted/published yet).

Poor data quality and/or lack of experimental data can impact the hazard assessment due to over- or underestimation of potential risks. For the assessed substances we did not find (high quality) data for all toxicological endpoints. For red phosphorous no data were located for carcinogenic, developmental and reproductive effects and for neurotoxicity a study of inadequate quality (inhalation exposure to aerosols of red phosphorous and butyl rubber mixture) was available; these endpoints were estimated to be of low hazard based on expert judgement (US-EPA, 2014a). In the MWCNT dossier no studies for reproductive effects were presented with the justification that MWCNTs would not become systemically available. Respiratory sensitisation is an endpoint where most substances have a data gap as there are no dedicated guideline studies to assess this endpoint. In case of data gaps, hazard is often estimated by analogy to structurally similar compounds for which experimental data are available and U.S.-EPA gives preference to the analogue approach over estimation. The type of analogues selected (strong analogues or not) can represent a source of uncertainty and may also impact the experts' conclusions. For instance, neurotoxicity of AlPi was estimated to be moderate by analogy to ATH in a GreenScreen® assessment. Recently, in vitro and in vivo studies have shown low neurotoxic potential for AlPi (Hendriks and Westerink, 2015).

Red phosphorous is obtained from the allotrope white phosphorous, which is much more reactive and toxic. Not distinguishing between phosphorous allotropes can lead to contradictory hazard conclusions for red phosphorus. For example in the U.S.-EPA DecaBDE alternative assessment the conclusion for acute toxicity changed from H to L following public consultation (US-EPA, 2014a). Uncertainties can be also due to the presence of pyrotechnic mixtures containing red phosphorous where toxicity cannot be referred to any specific component. In the DfE decisions based on expert judgement or studies carried out with analogues are marked by different font and colours.

In general, we found that REACH registration dossiers provide a comprehensive source of hazard and fate data which can be used for alternative hazard assessments. However, the dossiers contain the data the registrant selected and they may have not undergone any peer-review. The collected studies can be of varying quality and sometimes they are based on secondary sources and/or (very) old study reports with insufficiently detailed information. Not all information is publicly available, for example the substance identity may be confidential which could be important for the decision whether a selected study is relevant or not. Such specific information becomes particularly interesting when conflicting results for the same endpoint occur. Reliability scores (according to Klimisch et al. (1997)) can help to select the key studies and to support a conclusion based on weight of evidence. Our experience shows that the REACH dossiers are viable data sources whose content can be subject to continuous changes. Our conclusions are therefore only valid for the data that was available in mid-2016. Discrepancies in data availability and reliability make it challenging to choose the best alternative (Gilbertson and Ng, 2016, Hjorth et al., 2017).

For Deca-BDE a few GreenScreen® assessments were found. One suggested a benchmark of 1 (Rossi and Heine, 2007) based on the breakdown products penta-BDE and octa-BDE. No breakdown products were taken into consideration for other substances. In our case studies this may not make a difference, as the FRs investigated are all inorganic and recalcitrant to degradation, but in others it would.

When deriving the DNEL, the selection of the appropriate starting point and the AFs may influence the results considerably. In most cases no (sub)chronic inhalation study was available and chronic DNELs for inhalation were derived from oral studies by applying a route-to-route extrapolation. This assumes that the exposure route influences the effects only quantitatively (and this is corrected by different absorption routes) but may neglect that there could be qualitative differences. Inhaled particles basically induce local effects in the lung which are problematic to extrapolate from the oral route. Also for lung exposure the choice of the test system is relevant. Intratracheal instillation studies, as often carried out instead of inhalation studies with (nano)particles, may induce effects via mechanisms (overload) that are not relevant for human exposure situations (Oberdörster, 2015).

In the case of MWCNT the choice of method for the DNEL calculation has a major impact on the required exposure control. Strict application of the REACH default AFs leads to a DNEL in the range of 1–2 μg/m^3^ depending on whether the NOAEC or LOAEC of 1 mg/m^3^ is used as starting point (Aschberger and Christensen, 2010). This occupational exposure level lies in the range of the detection limit and is thus difficult to control. The REACH dossier suggests a DNEL of 50 μg/m^3^ based on a calculation by (Pauluhn, 2010b) (see Section 3.1.2). NIOSH considered interspecies differences in another manner and their calculations resulted in a level below 1 μg/m^3^. Finally they published a recommended exposure level of 1 μg/m^3^, based on the detection limit and the technical feasibility to control the exposure (NIOSH, 2013).

In the case of MWCNT it was also challenging to apply the thresholds for the hazard assignment according to DfE and GreenScreen®. The highest attainable dose in the acute inhalation study not leading to severe effects, did not match in the provided classes of DfE, as these are built on LC_50_ values. Likewise, in the GreenScreen® assessment the results from the chronic inhalation study could not be clearly assigned, resulting in a different benchmark score of 2 or 3. In general, hazard designations are determined by the most sensitive study (lowest concentration or most sensitive species). The spread of the available data which also gives important information on the toxicity profile of a substance is usually not considered (Gilbertson and Ng, 2016).

As shown above, uncertainties can be associated with each step of the assessment and need to be addressed. We have highlighted those that we encountered in our comparative hazard assessment and discussed them semi-quantitatively (Table 4).

We found ourselves confronted with very heterogeneous data and information from disparate sources. I addition when it comes to functional substitution (e.g. other flame retardant mechanism) instead of substitution of a chemical by one with similar chemical structure and the same/comparable function, the limits of the applied (simple) CAA tools and the need for methods that consider also the intrinsic exposure potential are demonstrated (Tickner et al., 2015, Gilbertson and Ng, 2016, Hjorth et al., 2017). The deployment of decision science into alternative analysis has been proposed to enable the incorporation and weighing of individual sources of information and uncertainty in the decision-making process (Linkov et al., 2013, Malloy et al., 2016).

## 4 Conclusions and discussion

To enable effective substitution of chemicals of concern, several methodologies have been proposed with the aim to assess and rank chemicals in a transparent and pragmatic way. Chemical alternatives assessments are a methodology for identifying, comparing and selecting safer alternatives to chemicals of concern. Most CAAs are designed to compare chemicals primarily based on hazards and provide users (producer, formulants, downstream user and consumer) with hazard based information thus allowing informed choices for selecting less hazardous chemical alternatives.

We have reviewed hazard and fate data for HFRs of concern (represented in this case study by DecaBDE) and potential substitutes and made an appraisal to compare and rank them based on these properties according to their DNELs and U.S.-EPA DfE and Greenscreen® scores. The selected approaches focus on human and environmental hazard as well as on the persistence and bioaccumulation potential. This review found that the most decisive property for substances of concern is bioaccumulation as this was the only property consistently judged throughout different evaluations especially for toxic substances without CMR property. It depicted the major difference between FRs of concern and their potential substitutes and is a property which is challenging to control. Persistence is an inherent property of many inorganic compounds, as they are recalcitrant to biodegradation and thus are persistent as stable moieties. As most alternative FRs are inorganic chemicals persistence alone cannot be a decisive factor unless it is combined with bioaccumulation and/or high toxicity. A comparison/ranking of exposure reference values such as DNELs showed that FRs of concern are not identified by a low DNEL.

Concerning toxicity we observed major differences between the various assessments with regard to the selection of toxicity studies, the interpretation of test results and the requirement to classify a substance according to CLP Regulation. This is particularly relevant for CMR properties, as this would exclude a FR from being suggested as safer alternative.

A major challenge for our assessment was to correctly identify the chemicals. In some cases only the trade names were given and we had difficulties to correctly assign one specific chemical to that trade name and to use the appropriate data. For some commercial FRs, which are mixtures and whose composition was not exactly known to us, we focused our assessment on the principal component.

From our experiences in carrying out chemical alternatives assessments we conclude:

i) REACH registration dossiers provide a comprehensive source of publicly available hazard and fate information which can be used for an alternative assessment. The quality of the presented data from industry is variable and the data are subject to changes without prior notice. Endpoint specific conclusions are sometimes based on secondary sources or old study reports with insufficiently detailed information (also concerning the substance identity) and have not been peer-reviewed e.g. by authorities. Not all data, including parts of the substance identity, are publicly available. These limitations need to be considered for any (automatic) data extraction used for alternative assessments, risk assessments, life cycle assessment or other purposes. Any user of the data is therefore encouraged to carefully compare the data against other sources and latest peer review literature.

ii) Correct identification of the chemicals is crucial to retrieve the appropriate data. For mixtures, reaction products or NMs or when only trade names are available this can be challenging. Also for the comparison of the chemicals the identity must be known, as otherwise the exercise is void.

iii) The quality of the data, the practice on how to fill data gaps can have a huge impact on the results and conclusions. When using data from analogues the similarity has to be well justified.

iv) Assessment criteria have mainly been developed for organic chemicals and pose challenges when applied to inorganic solids including NMs. Assessment criteria and hazard scores can change over time, when new data and/or knowledge become available or available data is interpreted in a different way. When searching for safer alternatives it is therefore important to not only consult the final benchmark score but also consider the data leading to these results.

v) Based on the challenges encountered, we consider it important to analyse and report uncertainties for each step of the decision making process.

We have based our assessment on publicly available data and selected publicly available transparent models which can be understood and applied also by non-scientists who have some knowledge on how to use and interpret the available hazard data. This approach has the limitation that the tools developed for conventional chemicals have been applied for nanomaterials for which there may not be a proper validation (Hjorth et al., 2017). This raises the question whether publicly available tools should be used by non-experts and if major conclusions can be made for nanomaterials or novel substances, if the validity range of the tools is not clearly indicated. Different evaluation systems may be needed to allow an efficient and effective comparison of new or heterogeneous substances or solutions to complex problems (Hjorth et al., 2017). To evidence all the uncertainties met in this alternative assessment, we followed the recommendations of the recently published uncertainty guideline (EFSA Scientific Committee, 2016).

We consider that this article provides an up-to-date repository of hazard and fate data of some FRs and describes relevant experiences when using such data. This information can be used as relevant contribution to comprehensive alternative assessments which furthermore include fire performance, life cycle considerations and socio-economic considerations to allow decisions on whether alternatives are satisfactory from a performance, economic, aesthetical and social point of view (Péry et al., 2013, NAS, 2014, Tickner et al., 2015, Malloy et al., 2016). Different stakeholders may profit from our experiences in carrying out such an assessment.

## Figures and Tables

**Table 1:** Ranking of flame retardants based on descending DNELs and hazard results (classification, endpoints of concern).

Substance	CAS/EC/list No	DNEL (μg/m^3^)^a^	Method – DNEL calculation	Hazard classification (CLP Regulation)^b^ (No of notification proposals if > 10)	(potential) endpoint of concern	Reference
Aluminium diethylphosphinate	List no: 607-114-5	60,000^+^	R2R extrapolation from oral study (NOAEL > 1000 mg/kg bw/d) AF: 30	Not classified (35)	U.S.-EPA: neurodevelopmental effects based on the presence of phosphinate	(ARCADIS, 2011, US-EPA, 2014a, US-EPA, 2015a) ^+^ (Exolit OP930 Dossier, 2016)
Aluminium trihydroxide	CAS-No: 21645-51-2; EC-No: 244-492-7	10,760	LOAEC of 33.5 mg/m^3^ AF: 9 (3 for LOAEL ➔ NOAL, 3 for intraspecies differences)	Not classified (1138); Skin Irrit. 2 H315; Eye Irrit. 2 H319; STOT SE 3 H335 (respiratory systemic) (228);	U.S.-EPA: Immunotoxicity GreenScreen®: Immunotoxicity Neurotoxicity Respiratory sensitiser	(ARCADIS, 2011, US-EPA, 2014a, US-EPA, 2015a, ATH Dossier, 2016)
*N*-alkoxy-hindered amine reaction products	CAS-No: 191680-81-6; EC-No 425-020-0	10,000	The general exposure limit for inhalable dust is applied	Not classified (72)	U.S.-EPA: repeated dose organ effects, reproductive and developmental effects, immunotoxicity; not supported by data in REACH dossier	(US-EPA, 2014a, CGL-16 Dossier, 2016)
Decabromodiphenyl ether (deca-BDE)	CAS-No: 1163-19-5; EC-No: 214-604-9	6000	R2R extrapolation from oral study (absorption rate 6% oral, 100% inhalation); AF: 25	Not classified (167); Acute Tox. 4 H302; Acute Tox. 4 H312; Eye Irrit. 2 H319; (20) Muta. 2 H341; STOT RE 2 H373 (liver, kidney); (14) Aquatic Chronic 4 H413; (12)	PBT Developmental effects Endocrine disruption	(EC DG ENV, 2000, US-EPA, 2014a, ECHA C&L inventory, 2016, SVHC, 2016)
Red phosphorus	List no: 918-594-3	4000	no N(L)OAEL available; no AF reported	Harmonised classification: Flam. Sol. 1 H228; Aquatic Chronic 3 H412;	Release of phosphine; generation of phosphoric acid containing smoke during fire	(US-EPA, 2014a, ECHA C&L inventory, 2016, Red Phosphorus Dossier, 2016)
MWCNT (NC7000, Baytubes®)	List no: 936-414-1	50	AF: 2 for rat-human differences in alveolar deposition, ventilation and time-dependent particle accumulation and clearance	Not classified (Dossier)	Repeated dose inhalation toxicity – NOAEC 1 mg/m^3^	(Pauluhn, 2010b, US-EPA, 2013, MWCNT Dossier, 2016)
MWCNT		1	Detection limit Default AF (REACH guidance); AF: 20/100	Not classified (list no 936-414-1) Carbon nanotube dispersion not further specified: Skin Irrit. 2 H315, Eye Irrit H319, STOT SE 3 H335 (respiratory system) carbon nanotube (CAS No 308068-56-6, EC No 308068-56-6) pre-registered: Eye Irrit H319, STOT SE 3 H335 (26)	Repeated dose inhalation toxicity: REL: 1 μg/m^3^	(Aschberger et al., 2010, NIOSH, 2013, ECHA C&L inventory, 2016)

EU-RAR: Risk Assessment report according to Regulation No 793/93.

CLP: Classification, Labelling and Packaging Regulation 1207/2008.

R2R: Route to route extrapolation.

NC: no classification.

STOT: specific target organ toxicity.

AF: Overall Assessment Factor (covering interspecies, intraspecies variability, duration).

C&L: classification and labelling.

SE: single exposure, RE: repeated exposure.

a For chronic worker exposure, as taken from REACH registration dossier.

b http://echa.europa.eu/information-on-chemicals/cl-inventory-database; European Parliament and Council (2008). Regulation (EC) No 1272/2008 of the European Parliament and of the Council of 16 December 2008 on Classification, Labelling and Packaging of Substances and Mixtures from http://eur-lex.europa.eu/LexUriServ/LexUriServ.do?uri=OJ:L:2008:353:0001:1355:en:PDF.

**Table 2:**
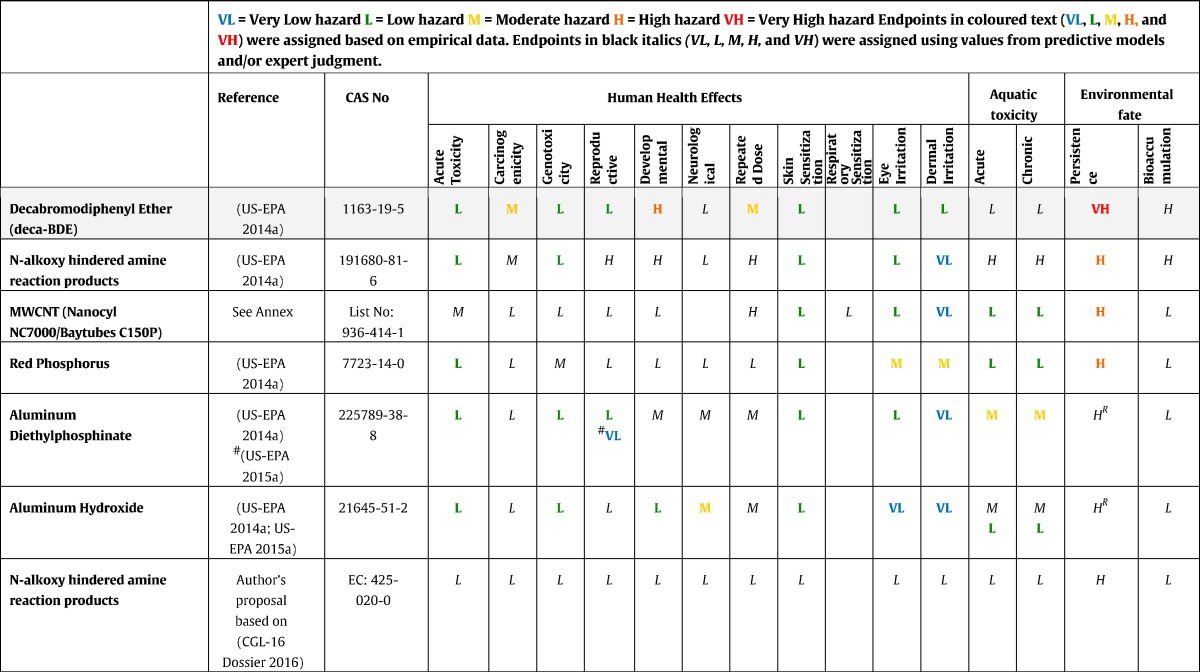
Screening level toxicity hazard summary of selected flame retardants (Design for Environment, U.S.-EPA).

**Table 3:**
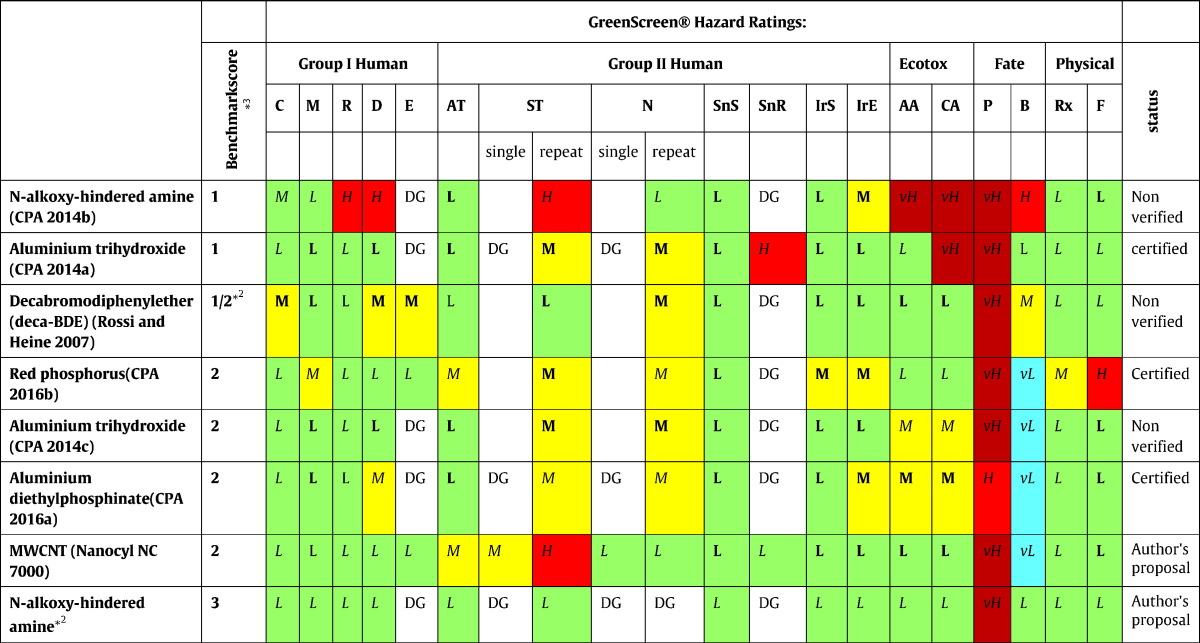
GreenScreen® Hazard Rating and benchmark score for selected flame retardants.

Note: Hazard levels (very high (vH), high (H), moderate (M), Low (L), very low (vL)) in *italics* reflect estimated values, authoritative B lists, screening lists, weak analogues, and lower confidence. DG = data gap.

*^1^ Available GreenScreen® evaluation gives a benchmark score of 2 for deca-BDE for Moderate Group II Human Health Effects, but based on breakdown products an overall Benchmark of 1 is assigned.

*^2^ Based on data provided in the REACH registration dossier.

*^3^ Benchmark scores: 1: Avoid – Chemical of high concern, 2: Use but search for safer substitutes, 3: Use, but still opportunity for improvement, 4: Prefer-Safer chemical.

**Table 4:** Appraisal of identified uncertainties and their potential impact on the results in the hazard alternative assessment.

Sources of uncertainty	Criteria to evaluate uncertainty	Flame retardant in case study	Impact on result in case study (Low-Medium High)	Explanation
*Data collection*				
Identity of the substance	IUPAC name, CAS No, EC No, list No (REACH registration). Detailed description of substance identity and phys-chem properties monoconsituent, pure substance variability/stability of composition in case of mixtures/UVCB	MWCNT	L	MWCNT types differ in: length, diameter, aspect ratio, surface modification, purity, catalysing impurities, rigidity; which may all have an impact on biological/environmental fate and effects. Our assessment focused on two similar, short tangled MWCNT types
		Aluminium diethylphosphinate (AlPi)	H	No registration dossier of AlPi available. Several commercial products (Exolit Opxxx) based on phosphinates such as AlPi are sold whose exact composition is not disclosed. REACH dossier on commercial product Exolit OP930 is mainly based on phosphinates such as AlPi.
		*N*-alkoxy hindered amine	H	Reaction product which could not reliably be assigned to a CAS number. Chemical described as Substances of Unknown or Variable composition. Complex reaction products or Biological materials (UVCB).
		Red phosphorus	M	Different allotropic forms of phosphorus exist with very different toxicity profile; Allotropes in studies not always specified. Only studies clearly disclosing identity should be consulted.
Completeness of data Management of data gaps Use of data with analogues/allotropes/categories	Number of reliable and relevant studies available per endpoint Justification of similarity (phys-chem, toxicokinetics, biological)	MWCNT	M	No studies available for carcinogenicity (inhalation), reproductive toxicity, respiratory sensitisation; As surrogate for inhalation intraperitoneal injection was used to assess carcinogenicity; Implicit grouping of two MWCNT types in the REACH dossier without further justification; data from other MWCNT types with similar size and phys-chem properties was used to fill data gaps; data from other MWCNT types only used when they strengthened the weight of evidence and the differences in phys-chem properties did not seem to have an impact on the biological effects.
		AlPi	M	Lack of neurotoxicity studies. Hazard estimation was based on structurally similar compounds for neurotoxicity (aluminium hydroxide) and neurodevelopmental effects (presence of phosphinates). Ongoing experiments could change neurotoxic potential estimation
		Aluminium trihydroxide (ATH)	H	Lack of data for several endpoints. Hazard estimation for several endpoints based on structurally similar compounds (i.e. acute toxicity, eye irritation, skin and respiratory sensitisation, carcinogenesis) with not sufficient scientific justification. Different conclusions by different evaluators for some endpoints.
		*N*-alkoxy hindered amine	H	Lack of carcinogenicity studies. In many cases hazard has been estimated by expert judgement where it is not clear which analogue compounds were used as surrogates.
		Red phosphorus	M	Lack of experimental data for several endpoints (i.e. carcinogenicity, reproductive and developmental effects, neurotoxicity). Some consulted studies do not distinguish between the presence of red phosphorous or its more toxic allotrope white phosphorous. There are also studies assaying mixtures containing red phosphorous.
		Deca-BDE	H	Depending on which study is selected as key study and how evidence is weighted, different conclusions with regard to neurodevelopmental toxicity can be drawn. This has a major impact on the toxicity hazard score. Many toxicity studies are not guideline studies.

*Data evaluation*				
Data quality (of key study selected) Experimental variability of results	Scientific plausibility (relevance for endpoint: exposure route, test system) Reliability: Klimisch score; Data quality: impact on weight of evidence	MWCNT	M	For most endpoint guideline studies (GLP) are available; several of these studies are unpublished reports and only the summary is publicly available.
			H	Genotoxicity tests showed low reproducibility (results from (Nanogenotox, 2013))
			M	Dose descriptors derived from rat studies may be of little relevance for humans when effects are a consequence of lung overload in rodents
		AlPi	M	Most information is based on secondary sources referring to confidential studies, whose content and quality could not be checked; as there is little discrepancy between conclusions on toxicological endpoints the uncertainty is not considered high
		ATH	H	Conclusions on neurotoxicity depend on key studies consulted; inconsistent experimental design, protocols and information on impurities;
Knowledge on breakdown products (and their consideration in the assessment)	Lower/higher hazard of breakdown products compared to parent compound	Deca-BDE	H	Breakdown products penta-BDE and octa-BDE are more toxic/bioaccumulating; including them in the assessment changes the hazard profile significantly
		MWCNT	L	Seems not relevant as it is pure carbon; the catalytic metals could be of relevance if dissolved
DNEL derivation (route to route extrapolation; assessment factors)	Choice of default or chemical specific adjustment factors (CSAF) CSAF have to be scientifically justified, to not underestimate the risk	MWCNT	H	Depending on how rat-human differences alveolar deposition, ventilation and time-dependent particle accumulation and clearance are evaluated, the DNEL can differ by a factor of 25–50; this has a major impact on the feasibility of exposure control.
		*N*-alkoxy hindered amine	L	Inhalation DNEL derived using the general exposure limit for inhalable dust. Calculated DNEL from oral study via route to route extrapolation leads to comparable result;
		Red phosphorous	L	In the REACH dossier no AF and no NOAEL is reported; as red phosphorus was estimated of low concern following repeated exposure (US-EPA, 2014a), the proposed DNEL seems sufficiently protective.
Interpretation of assessment criteria - consideration of nature and severity of effects	Assessment criteria (EPA-DfE, GreenScreen®; CLP criteria)	Deca-BDE	H	Inconsistent interpretation of results (e.g. neurodevelopmental toxicity) and diverging classification proposals for acute toxicity, skin irritation, mutagenicity can lead to significantly different hazard scores.
		MWCNT	H	Thresholds for DfE assignment seem not to work well with all type of limit doses - e.g. for acute studies when no LC_50_ can be reached
		ATH	M	Different interpretation of hazard data as L or M for immune- and neurotoxicity; Diverging classification proposals for: eye and skin irritation and respiratory sensitisation;

## Appendix A Supplementary data

Supplementary material Image 1

## Footnotes

1 http://echa.europa.eu/web/guest/candidate-list-table.

2 http://ec.europa.eu/environment/chemicals/endocrine/strategy/substances_en.htm#priority_list.

3 http://echa.europa.eu/documents/10162/dd1871fd-5cbf-4dd8-98dc-90ac4bd62017.

4 http://www.aoec.org/tools.htm.

5 EINECS 264-513-3; 71714-47-1; Bis(1,2,2,6,6-pentamethyl-4-piperidyl) ((3,5-bis(1,1-dimethylethyl)-4-hydroxyphenyl)methyl)butylmalonate.
