# Supplementary material for: Chemical alternatives assessment of different flame retardants – A case study including multi-walled carbon nanotubes as synergist
Source: Environ Int. 2017 Apr;101:27–45. doi: 10.1016/j.envint.2016.12.017 (PMC5357113; doi:10.1016/j.envint.2016.12.017)
Supplement: Supplementary file 1 — Supplementary material [file mmc1.docx]

**Part1:**

# Chemical Alternatives Assessment of Multi-walled carbon nanotubes following the criteria of U.S.-EPA Design for Environment^[[1]](#footnote-1)^

This Chemical Alternatives Assessment of MWCNT was prepared by following the criteria of the U.S. EPA (Environmental Protection Agency) Design for Environment ([US-EPA 2011](#_ENREF_47)). The data below refers to MWCNT as registered under REACH; list number 936-414-1 covering Nanocyl NC 7000 and Baytube C150P ([MWCNT REACH Dossier](#_ENREF_25)). The test material is specified when such information is available from the registration dossier, peer-reviewed literature or the dossier prepared by the OECD Working party on Manufactured Nanomaterials (WPMN) ([OECD; 2015](#_ENREF_32)). Information on the identity of the test materials from unpublished reports was available to the authors but cannot be disclosed in this document. Reliability (Rel) codes according to Klimisch ([Klimisch *et al.* 1997](#_ENREF_16)) were assigned as stated in the REACH ([MWCNT REACH Dossier](#_ENREF_25)) or OECD ([OECD; 2015](#_ENREF_32)) dossier. In case insufficient data with the two MWCNT types was available, results from studies with other types of MWCNT were considered for a weight of evidence evaluation.

**Table 1** Screening Level Hazard Summary of MWCNT LIST No 936-414-1 (according to Design for Environment, U.S.-EPA)

|  | **VL = Very Low hazard L = Low hazard M = Moderate hazard H = High hazard VH = Very High hazard Endpoints in coloured text (VL, L, M, H, and VH) were assigned based on empirical data. Endpoints in black italics *(VL, L, M, H,* and *VH*) were assigned using values from predictive models and/or expert judgment.** | | | | | | | | | | | | | | | |
| --- | --- | --- | --- | --- | --- | --- | --- | --- | --- | --- | --- | --- | --- | --- | --- | --- |
| **Chemical** | **CAS** | **Human Health Effects** | | | | | | | | | | | **Aquatic toxicity** | | **Environmental fate** | |
|  |  | **Acute Toxicity** | **Carcinogenicity** | **Genotoxicity** | **Reproductive** | **Developmental** | **Neurological** | **Repeated Dose** | **Skin Sensitization** | **Respiratory Sensitization** | **Eye Irritation** | **Dermal Irritation** | **Acute** | **Chronic** | **Persistence** | **Bioaccumulation** |
| MWCNT (Nanocyl NC7000/Baytubes C150P) | List number 936-414-1 | ***M*** | ***L*** | ***L*** | ***L*** | ***L*** |  | **H** | **L** | ***L*** | **L** | **VL** | **L** | **L** | **H** | ***L*** |

|  | MWCNT NC7000™  (Transmission electron microscopy; courtesy of Nanocyl SA) | **CAS No:** no |
| --- | --- | --- |
|  |  | **List No:** 936-414-1 |
|  |  | **MW:** ca 70,000,000 |
|  |  | **Physical forms:** solid |
|  |  | **Use:** flame retardant synergist |
| **SMILES:** C | | |
| **Synonyms:** Multi-walled carbon nanotubes (MWCNT), synthetic graphite in tubular shape; Nanocyl NC 7000; Baytube C150P | | |
| **Chemical Consideration:** Pure carbon based (>90%) inorganic compound; in the absence (scarcity) of experimental data, expert judgement using information from other MWCNT types was used. | | |
| **Polymeric:** No | | |
| **Metabolites, Degradates and Transformation Products:** None | | |
| **Analogues:** other types of MWCNT; Information from other MWCNT types was used, when found appropriate | | |
| **Structural Alerts:** not applicable; Length of MWCNT (fibre like shape) could be kind of alert | | |
| **Risk Phrases:** not classified by Annex VI Regulation (EC) No 1272/2008 (CLP) | | |
| **Hazard and Risk Assessment:** registered under REACH; substance will be evaluated under CORAP (Community rolling action plan)^[[2]](#footnote-2)^ | | |

| **PROPERTY/ENDPOINT** | | | | **DATA** | **REFERENCE** | **METHOD, DATA QUALITY, TEST MATERIAL** | | |  |  |  |
| --- | --- | --- | --- | --- | --- | --- | --- | --- | --- | --- | --- |
| **MWCNT: List number 936-414-1: Nanocyl NC 7000-Baytube C150P** | | | | | | | | |  |  |  |
| Melting Point (°C) | |  | | > 450°C (no pressure value reported)  No thermal decomposition up to 450°C observed | Study report 2007 ([MWCNT REACH Dossier](#_ENREF_25)) | Rel 1; EU Method A.1 | | |  |  |  |
| Boiling Point (°C) | |  | | > 450°C  No boiling point up to 450°C observed. Indication for ignition at 490°C. | Study report 2007 ([MWCNT REACH Dossier](#_ENREF_25)) | Rel 1; EU Method A.2 | | |  |  |  |
| Vapour Pressure  (mm Hg) | |  | | Not conducted as melting point > 300°C | ([MWCNT REACH Dossier](#_ENREF_25)) |  | | |  |  |  |
| Water Solubility (mg/L) | |  | | > 2 mg/L at 20°C,pH 7.5-9.2  Limit of quantification of the detection method (total dissolved carbon) | Study report 2007 ([MWCNT REACH Dossier](#_ENREF_25)) | Rel 1; OECD Guideline 105 | | |  |  |  |
| Log K_OW_ | |  | | not conducted, as substance is inorganic | Reference to REACH Annex XI  ([MWCNT REACH Dossier](#_ENREF_25)) |  | | |  |  |  |
| Flammability (Flash point) | |  | | Not flammable  Flashpoint not determined as substance is inorganic | Study report 2007 ([MWCNT REACH Dossier](#_ENREF_25)) | Rel 1; EU Method A10; EU Method A12, EU Method A.13; | | |  |  |  |
| Explosivity | |  | | Not explosive;  no exothermic reaction up to 450°C | Study report 2007 ([MWCNT REACH Dossier](#_ENREF_25)) | Rel 1; EU Method A.14;  Rel 2; VDI 2263; Part1 | | |  |  |  |
| Pyrolysis | |  | | Not flammable | Not specifically reported |  | | |  |  |  |
| pH | |  | | Not applicable | Scientifically not justified  ([MWCNT REACH Dossier](#_ENREF_25)) |  | | |  |  |  |
| pK_a_ | |  | | Not conducted as the substance does not contain any functional groups that may dissociate | ([MWCNT REACH Dossier](#_ENREF_25)) |  | | |  |  |  |
| Particle size | |  | | Thin and short tubular shape, thin tangled MWCNT (tube diameter d90 ≤ 30nm); length: 0.2 µm - 5 µm (calculated mean value; 1.5 µm; TEM and SEM) | ([MWCNT REACH Dossier](#_ENREF_25) ; [JRC-Repository; 2014](#_ENREF_14); [OECD; 2015](#_ENREF_32); [Nanocyl 2016](#_ENREF_28)) | (Baytubes C150P; Nanocyl NC 7000) | | |  |  |  |
| Specific surface area | |  | | 250-300 m^2^/g | (MWCNT REACH Dossier ; JRC-Repository; 2014; OECD; 2015; Nanocyl 2016) |  | | |  |  |  |
| Basic morphology | |  | | Thin, flexible, short and tangled | ([MWCNT REACH Dossier](#_ENREF_25)) |  | | |  |  |  |
| Granulometry | |  | | Tightly bound agglomerates consisting of tangled tubes. The particle size distribution of the substance, measured by digital microscopy, shows D10 = 597 µm, D50 = 842 µm and D90 = 995 µm. | ([MWCNT REACH Dossier](#_ENREF_25)) | (Baytubes C150P; Nanocyl NC 7000) | | |  |  |  |
| Purity:  Impurities | |  | | 90% Carbon  <5% Impurities  <3wt% Mn Mg, Al, Na, Ni, Fe;  >0.01%: Al, Co, Mg, Mn, Ca | ([Pauluhn 2010a](#_ENREF_33); [JRC-Repository; 2014](#_ENREF_14)) | ICP-OES (inductively coupled plasma optical emission spectrometry)  ICP-MS (inductively coupled plasma mass spectrometry)  (Baytubes C150P; Nanocyl NC 7000) | | |  |  |  |
| **HUMAN EFFECTS** | | | | | | | | |  |  |  |
| **Toxicokinetics** | | | | **MWCNT showed no extrapulmonary translocation following inhalation except to lung associated lymph nodes; there is no evidence for systemic availability after oral or dermal exposure.** | | | | |  |  |  |
| Dermal absorption *in vitro* | | | | No translocation through epidermis of 100 mg/ml applied topically for 24 - 48h on reconstructed human epidermis (RHE); measurements of viability of living cells and trans-epithelial-electrical resistance (TEER) showed no cytotoxicity.  No adverse effects and no evidence for systemic availability of MWCNT after dermal exposure in a guinea pig maximization test and in an acute dermal toxicity test in vivo and in vitro. | ([Vankoningsloo *et al.* 2010](#_ENREF_49))  Study reports (2006, 2007) ([MWCNT Dossier 2016](#_ENREF_24)) | Rel 2; | | |  |  |  |
| Absorption, Distribution, Metabolism&Excretion | | Inhalation | | Rat Inhalation: 0.1, 0.4, 1.5, 6 mg/m^3^ (6h/d, 7d/w, 90 days, recovery period 6 months): time and concentration dependent clearance from lung and translocation to lung associated lymph nodes (LALN) after 13weeks (tracer Co); marked inhibition of clearance and higher biopersistence at higher concentration (i.e. 1.5 and 6 mg/m^3^ due to overload conditions). No extrapulmonary translocation to other organs of maker cobalt; | ([Ellinger-Ziegelbauer and Pauluhn 2009](#_ENREF_6); [Pauluhn 2010a](#_ENREF_33)) | Rel 1; OECD TG 413 (Baytubes C150P);  Rel 1; OECD TG 403 (Baytubes C150P); | | |  |  |  |
|  |  |  |  | Rat intratracheal instillation (0.5 mg/animal once): Rapid clearance (ground substance 36% after 60 days) | ([Muller *et al.* 2005](#_ENREF_20)) | Rel 2 (Nanocyl NC 7000); | | |  |  |  |
|  |  | Oral | | After oral administration ^14^C-MWCNT (10 mg/kg bw (bodyweight) oral gavage once or during 5 consecutive days) did not show translocation from the GI-tract into the systemic circulation or any of the organs investigated (including spleen, liver, and lung). | ([Jacobsen *et al.* 2013](#_ENREF_13)) | Rel 1 (Nanocyl NC 7000); | | |  |  |  |
|  |  |  |  | Rat oral gavage (0.05, 0.5 mg/kg bw for 28 d): urinary excretion; granulomatous changes in liver and MWCNT in urine, without findings of MWCNT in liver and kidneys. | Study report 2008 ([MWCNT Dossier 2016](#_ENREF_24)) | Rel 2 | | |  |  |  |
|  |  |  |  | In vitro permeability study(Caco-2 cells): no penetration | Study report 2010 ([MWCNT Dossier 2016](#_ENREF_24)) | Rel 2 | | |  |  |  |
|  |  |  |  | Lack of toxicity observed in animal treated with oral doses indicates that either no or a very low absorption after oral exposure is expected. | ([Binderup *et al.* 2013](#_ENREF_1)) |  | | |  |  |  |
|  |  | Injection | | After intravenous (IV). injection of ^14^C-MWCNT (10 mg/kg bw once or during 5 consecutive days) most of the injected dose was observed in liver (24%) and lung (25%) at day 1, with a few percent present in spleen, kidneys, heart and testes. At day 90, 20% of injected dose is still observed in organs as compared to 65% observed at day 1 (mainly liver and lung). | ([Jacobsen *et al.* 2013](#_ENREF_13)) | Rel 1 (Nanocyl NC 7000);  Nanogenotox project | | |  |  |  |
| **Acute Mammalian Toxicity** | | | | **LOW: Experimental studies indicate that oral and dermal routes to rats do not produce substantial mortality up to the limit doses (oral LD 50 ≥ 5000 mg/kg bw; dermal LD 50 > 2000 mg/kg bw);**  ***MEDIUM*: MWCNT are estimated of medium hazard for acute inhalation. No lethality and only mild effects were observed at the highest technically attainable concentration at a very low concentration (inhalation LC_50_ > 0.241 mg/L).** | | | | |  |  |  |
| Acute Lethality | | Oral | | Rat Oral (feed) LD_50_ ≥ 5000 mg/kg bw (no clinical signs) | Study report 2006 ([MWCNT Dossier 2016](#_ENREF_24)) | Rel 1, OECD TG 423 | | |  |  |  |
|  |  |  |  | Rat Oral (gavage); liver toxicity > 100 mg/kg bw (no dose dependent granulomatous changes in the liver 24 hours after a single dosage of 0.5, 1.5, 50, 100 mg/kg) | Study report 2008 ([MWCNT Dossier 2016](#_ENREF_24)) | Rel 2; OECD TG 420 | | |  |  |  |
|  | | Dermal | | Rat Dermal LD_50_ > 2000 mg/kg bw (no clinical signs) | Study report 2006 ([MWCNT Dossier 2016](#_ENREF_24)) | Rel 1; OECD TG 402 | | |  |  |  |
|  | | Inhalation | | Rat Inhalation (nose only) LC_50_ (6h)> 241 mg/m^3^ air (maximum technically attainable concentration); no deaths and only transient clinical signs (irregular and laboured breathing patterns and reduced body weights)  Inhalation NOAEC: 11 mg/m^3^ air  The highest tested dose is lower than lowest threshold for hazard assignation based on LC_50_ value (< 0.5 mg/L/d, very high), therefore the criteria do not seem to be applicable for that substance. Effects at the highest possible concentration were mild, thus a hazard assignation of very high or high does not seem to be justified, as a concentration inducing severe effects is not expected to occur. No classification according Regulation 1207/2008 is proposed. | ([Ellinger-Ziegelbauer and Pauluhn 2009](#_ENREF_6)) | Rel 1; OECD TG 403 (Baytubes C150P) | | |  |  |  |
| **Carcinogenicity** | | ***LOW*: This type of MWCNT is estimated of low hazard for carcinogenicity. No tumours were observed following intraperitoneal injection of short, tangled, low-density agglomerate form of MWCNT; IARC: cat. 3 (not classifiable as to its carcinogenicity to humans); MWCNT of more rigid, long-fibre (asbestos-like) morphology were classified as possibly carcinogenic to humans (Group 2B).** | | | | | | |  |  |  |
|  | | OncoLogic Results | |  |  |  | | |  |  |  |
|  | | Carcinogenicity (Rat)  (intraperitoneal injection) | | Rat 2 year bioassay following single intraperitoneal injection of 2 and 20 mg/animal. MWCNT with or without structural defects did not induce mesothelioma in this bioassay. The incidence of tumours other than mesothelioma was not significantly increased across the groups. | ([Muller *et al.* 2009](#_ENREF_23)) | Rel 2; (Nanocyl NC 7000) | | |  |  |  |
|  | |  | | Rat: 2 injections of 5 mg with one-week interval. Exposure 1 year and over life time (~3 years).  Short tangled MWCNT did not induce mesotheliomas after one and three years; negligible chronic inflammation and macrophage activation after 1 year. Induction of granulomas (without iron deposition) and various types of tumours (e.g. adrenal gland cortical hypertrophy, mammary gland hyperplasia, bile duct cell tumour) mostly associated with physiological senility. | ([Nagai *et al.* 2011](#_ENREF_26); [Nagai *et al.* 2013](#_ENREF_27)) | Rel 2: small number of animals tested over life time: 6 male and 9 female for 1 year.  (other type: NTtngl: tangled MWCNT, 15 nm diameter and 3000 nm length) | | |  |  |  |
|  | | Carcinogenicity (Rat)  (intraperitoneal/interscrotal injection) | | Studies with MWCNT of more rigid, long-fibre (asbestos-like) morphology (Mitsui MWNT-7; length: 5.0+/-4.5 µm; diameter: 40-90 nm) showed positive results:  1) single intraperitoneal injection of 3 mg p53+/-mouse; postexposure: up to 25weeks; Induction of mesothelioma and high lethality observed;  2) single intraperitoneal injection of 3, 30, 300 µm in p53+/- mouse; postexposure up to 1year; Observation of dose dependent but obviously time independent onset of peritoneal adhesion and granuloma formation (5/20, 17/20, 19/20 mesotheliomas);  3) single interscrotal injection of 1 mg/kg-bw in rat; postexposure: 52w, 6/7 rats died/moribund due to mesothelioma at week 37-40; mesothelioma and metasthasis in other organs including pleura; 1 rat survived without mesothelioma. | ([Takagi *et al.* 2008](#_ENREF_44))  ([Takagi *et al.* 2012](#_ENREF_45))  ([Sakamoto *et al.* 2009](#_ENREF_41)) | (other type of MWCNT)  Rel 2  Rel 2  Rel 2 | | |  |  |  |
|  | | Carcinogenicity (Rat)  (inhalation, intratracheal instillation) | | 1) long, rigid type of MWCNT (Mitsui MWNT-7; length: 5.0+/-4.5 µm; diameter: 40-90 nm)  Inhalation exposure to MWCNT (5 mg/m³, 5 hours/day, 5 days/week) for 15 days one week after intraperitoneal injections of tumour initiator methylcholanthrene. After 17 months post-exposure, MWCNT exposures promoted the growth and neoplastic progression of initiated lung cells. The lung burden of 31.2 μg/mouse is estimated to approximate feasible human occupational exposure.  2) MWCNT of comparable dimensions: (Nikiso: length: length: 1 µm; diameter: 50 nm)  Intratracheal instillation of 1 mg/rat during the initial 2 weeks of the experiment and then observed up to 109 weeks. Induction of malignant mesothelioma and lung tumours in rats. | ([Sargent *et al.* 2014](#_ENREF_42))  ([Suzui *et al.* 2016](#_ENREF_43)) | (other type of MWCNT) | | |  |  |  |
|  | | Parameters influencing carcinogenicity | | Important factors and crucial step for the formation of mesothelial carcinogens are the clearance from lung, the entry into and clearance from mesothelium. These depend on solubility/durability, length, diameter and shape. All MWCNT are biopersisitent. The longer and more rigid/needle like a MWCNT/fibre the higher the risk, the shorter and the more bent, curved or waved the lower their toxic and carcinogenic potency seems to be.  In 2014, the International Agency for Research on Cancer (IARC) classified MWCNT-7 (Diameter: 74 ± 28 nm, length: 5.7 ± 3.7 µm) as possibly carcinogenic to humans (Group 2B) based on the evidence of observed mesothelioma following intraperitoneal (or intrascotal) injection in rats. All other SWCNTs and MWCNT were categorised in Group 3 as not classifiable as to their carcinogenicity to humans. | ([Donaldson *et al.* 2010](#_ENREF_5); [Rittinghausen *et al.* 2014](#_ENREF_39))  ([Grosse *et al.* 2014](#_ENREF_10)) |  | | |  |  |  |
|  | | Combined Chronic Toxicity/Carcinogenicity  (inhalation) | | Rat 90 day inhalation (+6months postexposure): NOAEC 0.1 mg/m^3^ (Baytubes);  Rat 90 day inhalation LOAEC 0.1 mg/m^3^ (NC 7000):  Both granulomatous inflammation, no (pre)neoplastic formations | ([Ma-Hock *et al.* 2009](#_ENREF_19); [Pauluhn 2010a](#_ENREF_33)) | Rel 1; OECD TG 413;  (Nanocyl NC 7000; Baytubes C150P) | | |  |  |  |
| **Genotoxicity** | | | | ***LOW:* MWCNT are estimated of low hazard for genotoxicity; the majority of *in vitro* studies negative; *in vivo* studies were negative except one with slightly positive effects considered not reliable due to methodological deficiencies. Observed positive responses were low and may represent secondary genotoxicity following oxidative stress.** | | | | |  |  |  |
| Gene mutation *in vitro* | | | | Negative in Ames test up to 5000 μg/plate (with/without metabolic activation)  Negative in Ames test up to 2000 μg/plate (with/without metabolic activation) | ([Wirnitzer *et al.* 2009](#_ENREF_51))  Study report 2010 in ([MWCNT Dossier 2016](#_ENREF_24)) | Rel 1; OECD TG 471 (Baytubes C150P)  Rel 1; OECD TG 471 | | |  |  |  |
|  |  |  |  | Negative in Chinese Hamster lung fibroblasts V79up to 100 μg/ml (with/without metabolic activation) | Study Report 2010 in ([MWCNT Dossier 2016](#_ENREF_24)) | Rel 1, OECD TG 476 | | |  |  |  |
|  |  |  |  | Negative in mouse lymphoma cell L5178Y-TK (4-32 ug/ml); | ([Nanogenotox 2013](#_ENREF_30)) | No Rel stated (Baytubes C150P, Nanocyl NC7000) | | |  |  |  |
| Gene mutation *in vivo* | | | | - |  |  | | |  |  |  |
| Chromosomal Aberration/Micronucleus *in vitro* | | | | Negative in Chinese Hamster lung fibroblasts V79; 2.5-10 μg/ml for 18 h (with/without metabolic activation) | ([Wirnitzer *et al.* 2009](#_ENREF_51)) | Rel 1, OECD TG 473 (Baytubes C150P) | | |  |  |  |
|  |  |  |  | Negative in Micronucleus test and Comet assay in A549 human pulmonary epithelial cells; ; 2.8-11.25 and 7.5 – 30 μg/ml for 24 and 72 h respectively (with/without metabolic activation) | ([Thurnherr *et al.* 2011](#_ENREF_46)) | REL 3 (no validated cell line, no appropriate positive, negative controls) (Baytubes C150P) | | |  |  |  |
|  |  |  |  | Micronucleus assay (in vitro cytokinesis-block) in  - rat lung epithelial cells; 25 μg/ml for 24h and 10 - 50 μg/ml for 12 or 24h (with/without metabolic activation)  – significant differences of micronucleated cells; results not confirmed in second independent assay  - human breast carcinoma cells (MCF-7) 10 - 50 μg/ml for 12 or 24h (with/without metabolic activation)  – significant differences of micronucleated cells as sub-cytotoxic doses – second experiment: no dose-response relationship | ([Muller *et al.* 2008a](#_ENREF_21))  ([Muller *et al.* 2008b](#_ENREF_22)) | Rel 3 (no validated cell lines, no positive, negative controls, no assessment of cytotoxicity in RLE, cytotoxicity in MCF7)  (probably Nanocyl NC 7000 – not clear from the publication) | | |  |  |  |
|  |  |  |  | Micronucleus assay (0 – 20/250 μg /ml for 24 h) in  bronchial epithelial cells (BEAS-2B), human bronchial epithelial cells (16 HBE), human pulmonary epithelial cells (A459) and human epithelial colorectal adenocarcinoma cells (Caco2):  positive results in 3 out of 6 labs for Baytubes ® in Caco2 cells (two negative, one equivocal), and in BEAS 2B cells in 1 lab, while negative in 5 labs. Baytubes were also positive in human lymphocytes, but negative in 16 HBE and A549.  Nanocyl showed increased MN frequency in BEAS-2B, A549 and Caco2 cells, while it was negative in 16-HBE.  Positive results were weak and interpreted as consequence of oxidative stress, probably due to catalytic metals. | ([Vales *et al.* 2016](#_ENREF_48)),  ([Nanogenotox 2013](#_ENREF_30)) | Rel 1¸ OECD TG 487  (Baytubes C150P, Nanocyl NC7000) | | |  |  |  |
|  |  |  |  | Micronucleus assay (1, 10, 20 μg/ml for 28 d) in BEAS-2B cells; Significant increases in the levels of chromosome damage (micronuclei) at 10 μg/ml at 24 and at 20 μg/ml after 1 week and 3 weeks. Increased levels of intracellular reactive oxygen species (ROS) seems to be associated to solubilized metals contaminants. | ([Vales *et al.* 2016](#_ENREF_48)) | No Rel stated (Baytubes C150P) | | |  |  |  |
| DNA damage (Comet assay) | | | | Comet assay (0 – 20/250 μg /ml for 24h, 3d); in  bronchial epithelial cells (BEAS-2B), human bronchial epithelial cells (16 HBE), human pulmonary epithelial cells (A459) and human epithelial colorectal adenocarcinoma cells (Caco2):  were all negative for Baytubes® and NC7000 | ([Nanogenotox 2013](#_ENREF_30)) | No Rel stated (Baytubes C150P, Nanocyl NC7000) | | |  |  |  |
|  |  |  |  | Comet assay (1, 10, 20 μg/ml for 28 d) in BEAS-2B cells; negative (no primary damage) | ([Vales *et al.* 2016](#_ENREF_48)) | No Rel stated, Baytubes C150P | | |  |  |  |
| Chromosomal Aberration/Micronucleus *in vivo* | | | | Rat, oral gavage: 3.2, 6.4, 12.8 mg/kg/d for 3 days (24h interval) test after 3-6h; no significant increase of micronuclei, no change in ratio of polychromatic to normochromatic erythrocytes (PCE/NCE) in bone marrow and colon | ([Nanogenotox 2013](#_ENREF_30)) | No Rel stated (Nanocyl NC 7000) | | |  |  |  |
|  |  |  |  | Rat, intratracheal instillation: 0.12, 0.24, 0.48 mg/kg/d for 3 days (24h interval) test after 3-6h; no significant increase of micronuclei, no change in ratio of polychromatic to normochromatic erythrocytes (PCE/NCE) in bone marrow and colon | ([Nanogenotox 2013](#_ENREF_30)) | No Rel stated (Nanocyl NC 7000) | | |  |  |  |
|  |  |  |  | Rat, oral gavage: 0.05, 0.5 mg/kg for 28 days; Slight (not statistically significant) increased of micronucleated reticulocytes | Study report 2010 in ([MWCNT REACH Dossier](#_ENREF_25)) | Rel 4 (methodological deficiencies)  (Nanocyl NC 7000) | | |  |  |  |
|  |  |  |  | Rat, intratracheal instillation: 0.5, 2, 5 mg/rat; No dose dependent increase of nucleated AT-II-cells (ex vivo) in bronchoalveolar lavage; significant increase at 9.1 mg/kg | ([Muller *et al.* 2008a](#_ENREF_21)) | Rel 3 (methodological deficiencies: no standard protocol, no validated cell line, lack of historical data o cell line and cell viability, no valid positive control)  (Nanocyl NC 7000) | | |  |  |  |
|  |  |  |  | Rat, oral gavage: 3.2, 6.4, 12.8 mg/kg/d for 3 days (24h interval) test after 3-6h; no statistically significant increase in DNA in tail in liver, colon, kidney and spleen except in kidney at lowest dose with FpG (3.2 mg/kg/d) - results considered equivocal; great inter-animals heterogeneity | ([Nanogenotox 2013](#_ENREF_30)) | (Nanocyl NC 7000) | | |  |  |  |
| DNA damage (Comet assay) *in vivo* | | | | Rat, intratracheal instillation: 0.12, 0.24, 0.48 mg/kg/d for 3 days (24h interval) test after 3-6h; no significant increase of DNA tail in liver, kidney and spleen, except kidney at highest dose 0.48 mg/kg/d in absence of FpG | ([Nanogenotox 2013](#_ENREF_30)) | (Nanocyl NC 7000) | | |  |  |  |
|  |  |  |  | Rat, intratracheal instillation: 25,6 μg/w for 5weeks in ApoE KO-mice; no significantly increased level of DNA strand breaks in lung | ([Cao *et al.* 2014](#_ENREF_3)) | (Nanocyl NC 7000) | | |  |  |  |
| **Reproductive Effects** | | | | ***LOW*: MWCNT are estimated of low hazard for reproductive effects based on expected low systemic availability and no indications from subchronic inhalation studies and prenatal developmental toxicity studies. Reproductive toxicity studies with other MWCNT via physiological routes of exposure did not indicate to reproductive/developmental effects. Effects were seen with other MWCNT types at concentrations causing maternal toxicity when using non-physiological routes of exposure. Chronic inflammation could interfere with reproductive parameters.** | | | | |  |  |  |
| Reproduction and Fertility Effects | |  | | Low potential for reproductive effects;  Estimation based on expert judgment: lack of systemic availability; no effects in female and male reproductive organs in subchronic inhalation studies up to concentrations that caused lung toxicity. | ([MWCNT REACH Dossier](#_ENREF_25)) based on: ([Ma-Hock *et al.* 2009](#_ENREF_19); [Pauluhn 2010a](#_ENREF_33)) | Estimation based on study: OECD 413; (Nanocyl NC 7000, Baytubes CP 150) | | |  |  |  |
|  | |  | | A literature review of "Reproductive and developmental toxicity of carbon-based nanomaterials" reported in mice foetal malformations after intravenous and intraperitoneal injections and intratracheal instillation, foetal loss after intravenous injection, behavioural changes in offspring after intraperitoneal injection.  These effects occurred at rather high doses or not dose-dependent using physiologically non relevant routes of exposure and in the presence of maternal toxicity, when this was reported.  Oxidative stress was discussed to be implicated in foetal effects and that mutations in foetal tissues may be a consequence of transplacental mutagenesis.  Oral administration of MWCNT to dams was not associated with adverse effects on foetal development in rats and female reproduction and growth of offspring in mice | ([Ema *et al.* 2016](#_ENREF_8)) | Different types of CNT | | |  |  |  |
|  | | Reproduction/Developmental toxicity Screen | | Mouse, intratracheal exposure; single dose of 67 µg pre-mating,  Long lasting pathological changes (up to 4 months) in dam lung (inflammation) and liver (increased number of Kupffer cells, hypertrophy, hyperplasia), short delayed in delivery of first litter; no changes in gestational or offspring parameters. Lung inflammation due to particle exposure could interfere with female reproductive parameters (release of cytokines, acute phase proteins and other signalling molecules). | ([Hougaard *et al.* 2013](#_ENREF_11)) | Rel 2; (Nanocyl NC7000) | | |  |  |  |
|  | | Combined Repeated dose with Reproduction/Developmental Toxicity Screen | | Rat, oral feed 0, 1200, 3600, 12000 mg/kg food, two weeks before mating, during mating period and until day 4 post-partum (female). Sacrifice of females 4 d post-partum and males 28 d after treatment.  NOAEL (parental, reproductive and developmental) at the high (limit) dose level of 11.500 mg/kg food. This corresponds to the following actual substance intake: 813 mg/kg body weight/day for males; 1067 mg/kg body weight/day for females in the premating period, 930 mg/kg body weight/day for females during gestation and 1159 mg/kg body weight/day for females during lactation, respectively. | Study report (2010) in ([Graphite REACH Dossier](#_ENREF_9)) | Rel 1; OECD Guideline 422 (Combined Repeated Dose Toxicity Study with the Reproduction / Developmental Toxicity Screening Test)  Other MWCNT type (Graphistrength; length ~4 μm) | | |  |  |  |
| **Developmental Effects** | | | | ***LOW*: MWCNT are estimated of low hazard for developmental effects based on expected low systemic availability and no indications from subchronic inhalation studies and prenatal developmental toxicity studies. Developmental toxicity studies with other MWCNT via physiological routes of exposure did not indicate to reproductive/developmental effects. Effects were seen with other MWCNT types at concentrations causing maternal toxicity when using non-physiological routes of exposure. Chronic inflammation could interfere with reproductive parameters.** | | | | |  |  |  |
| Developmental effects | | | | Low potential for reproductive effects  Estimation based on expert judgment: lack of systemic availability; no effects in female and male reproductive organs in subchronic inhalation studies up to concentrations that caused lung toxicity. | ([MWCNT REACH Dossier](#_ENREF_25)) based on: ([Ma-Hock *et al.* 2009](#_ENREF_19); [Pauluhn 2010a](#_ENREF_33)) | Estimation based on study: OECD 413; (Nanocyl NC 7000, Baytubes CP 150) | | |  |  |  |
|  |  |  |  | A literature review of "Reproductive and developmental toxicity of carbon-based nanomaterials" reported in mice fetal malformations after intravenous and intraperitoneal injections and intratracheal instillation, fetal loss  after intravenous injection, behavioral changes in offspring after intraperitoneal injection.  These effects occurred at rather high doses or not dose-dependent using physiologically non relevant routes of exposure and in the presence of maternal toxicity, when this was reported.  Oxidative stress was discussed to be implicated in foetal effects and that mutations in fetal tissues may be a consequence of transplacental mutagenesis.  Oral administration of MWCNT to dams was not associated with adverse effects on foetal development in rats and female reproduction and growth of offspring in mice | ([Ema *et al.* 2016](#_ENREF_8)) | Different types of CNT | | |  |  |  |
|  | | Prenatal Development | | Rat oral gavage of rats (gestation day 6 through 19); 0, 40, 200, and 1,000 mg/kg/day;  Minimal maternal toxicity (decrease in thymus weight ) and no embryo–foetal toxicity at 1,000mg/kg/day;  NOAEL: 200 mg/kg/day for dams and 1,000 mg/kg/day for embryo–foetal development. | ([Lim *et al.* 2011a](#_ENREF_17); [Lim *et al.* 2011b](#_ENREF_18)) | Rel 4, OECD 414 (Prenatal Development Toxicity Study)  Other MWCNT type (Hanwha CM-95; length ~20 μm) | | |  |  |  |
|  | | Prenatal Development (pre-conceptional exposure) | | Mouse, intratracheal exposure; single dose of 67 µg pre-mating; or 4x 67 µg at gestation day 8, 11, 15 and 18;  Long lasting pathological changes (up to 4 months) in dam lung and liver, short delay in delivery of first litter; no changes in gestational or offspring parameters (e.g. behaviour, sperm quality in male offspring). Lung inflammation due to particle exposure could interfere with female reproductive parameters. The actual exposure to contaminants was considered too low to have influenced the delay in delivery of the first litter. | ([Hougaard *et al.* 2013](#_ENREF_11)) | Rel 2, (Nanocyl NC7000) | | |  |  |  |
| **Neurotoxicity** | | | | **No conclusion due to lack of data** | | | | |  |  |  |
|  | | Neurotoxicity Screening Battery (Adult) | |  |  | | | |  |  |  |
|  | | other | |  |  | | | |  |  |  |
| **Repeated dose Effects** | | | | **HIGH: MWCNT induced sustainable lung inflammation as reaction to lung overload; Due to the low NOAEC/LOAEC of 0.1 mg/m^3^, (= 0.00001 mg/L) MWCNT are of considered of high hazard; No oral and dermal study available (not considered as relevant exposure route) but risks are estimated low due to lack of translocation/absorption via these routes;** | | | | |  |  |  |
|  | | Inhalation | | Rat 90 day inhalation study (nose only exposure, 6h/d, 5d/w)  1) 13 weeks exposure to 0.1, 0.4, 1.5, 6 mg/m^3^ Baytubes CP150, including a 6 months post-exposure period. Overload associated pulmonary inflammation at high exposure concentration (1.5, 6 mg/ m^3^) which was not reversible within recovery period of 6 months. Translocation of MWCNT into lung associated lymph nodes was detectable only after 13 weeks and sustained elevations in neutrophylic granulocytes in the bronchoalveolar lavage occurred at the two highest concentrations with borderline effects at 0.4 mg/m3. No systemic toxicity in major organs (liver, kidney, heart); was detected at any of the concentrations tested.  At the lowest tested concentration of 0.1 mg/m^3^ all endpoints examined (based on nasal and pulmonary responses) were unchanged from the control treatment group, and this concentration was suggested as a NOEC (no observed effect concentration).  2) 13 weeks exposure to 0.1, 0.5, 2.5 mg/m^3^ Nanocyl NC 7000 not including a post-exposure period.  This study confirms absence of any pathological response in major organs such as the liver, kidney or heart and confirms in principle the findings of adverse pulmonary effects. Increased lung weights, pronounced multifocal granulomatous inflammation, diffuse histiocytic and neutrophilic inflammation, and intra-alveolar lipoproteinosis were observed in lung and lung-associated lymph nodes at 0.5 and 2.5 mg/m^3^. These effects were accompanied by slight blood neutrophilia at 2.5 mg/m^3^. The incidence and severity of the effects were concentration-related. At the lowest exposure level, 0.1 mg/m^3^, minimal granulomatous-type inflammation in the lungs and lung-associated lymph nodes were observed and this concentration was considered a LOEC (lowest observed effect concentration).  NOAEC/LOAEC: 0.1 mg/m^3^  3) In a 90 day inhalation study carried out with a different type of MWCNT (Graphistrength) lung inflammation characteristic of an overload with insoluble particles was observed after exposure to 5.0 mg/ m^3^. The NOAEC was 0.1 mg/m^3^.  None of the other inhalation studies in the OECD dossier with different types of CNTs reported a lower NOAEC/LOAEC than 0.1 mg/m^3^.  MWCNT of different physicochemical properties (small and large) caused similar inflammatory responses, but differences in transcriptional and histological markers of fibrosis in mouse lungs. | ([Pauluhn 2010a](#_ENREF_33); [Pauluhn 2010b](#_ENREF_34))  ([Ma-Hock *et al.* 2009](#_ENREF_19))  ([Pothmann *et al.* 2015](#_ENREF_36))  ([OECD; 2015](#_ENREF_32))  ([Poulsen *et al.* 2015](#_ENREF_37)) | Rel 1;OECD TG 413 (studies 1-3) | | |  |  |  |
|  | | Oral exposure | | Rat oral (gavage): 0.05 – 0.5 mg/kg bw/d for 28 day: observed liver toxicity (presence of inflammatory granulomatous changes in the hepatic parenchyma) was not dose-dependent. Histological analysis did not show changes in: oesophagus, stomach, small and large intestine, mucous membrane of the gastro-intestinal tract, spleen, pancreas and kidneys (cortex and medullar). | Study report (2008) ([MWCNT REACH Dossier](#_ENREF_25)) | Rel 2; On basis of OECD TG 407; (study disregarded, because of dose selections and the number of dose groups it was not possible to draw conclusions) | | |  |  |  |
|  | | Dermal exposure | | No information available, not a relevant route of exposure |  |  | | |  |  |  |
|  | | Immune System Effects | | No information available |  |  | | |  |  |  |
| **Skin Sensitisation** | | | | **LOW: MWCNT is not a skin sensitiser** | | | | |  |  |  |
|  | | Skin Sensitisation | | Not sensitising to guinea pigs in an in vivo maximisation test.  Other MWCNT types tested in a TG 406 or TG 429 (Local Lymph Node Assay) did not point to any sensitising potential. | Study report (2007) ([MWCNT REACH Dossier](#_ENREF_25))  ([Graphite REACH Dossier](#_ENREF_9) ; [Ema *et al.* 2011](#_ENREF_7)) | | Rel 1; OECD TG 406 | |  |  |  |
| **Respiratory Sensitisation** | | | | ***LOW*: MWCNT has low potential to be a respiratory sensitiser** |  | | |  |  |  |  |
|  | | Respiratory Sensitisation | | No information available for NC7000 and Baytubes.  In a mouse asthma model experiment testing a similar type of CNTs (Graphistrength) a dose-dependent increase of systemic immune response, as well as airway allergic inflammation and remodelling induced by house dust mite in the mouse was observed. MWCNT were administered via intratracheal instillation in doses of 75 μg MWCNT (25 μg/week) or 225 μg MWCNT (25 μg every other day). MWCNT did not induce significant effects, except that the highest concentration affected mucus production and increased thymic stromal lymphopoietin and interleukin-25.  MWCNTs have shown to promote allergic immune responses in mice following intranasal administration.  MWCNTs  MWCNTs displayed to exacerbate airway inflammation in mice using intratracheal instillation.  Carboxylic functionalised MWCNTs (c-MWCNT) in an in vitro study did not affect the ability of dendritic cells to activate T-lymphocytes to initiate an immune responses (Wang et al., 2009). | ([Ronzani *et al.* 2014](#_ENREF_40))  ([Nygaard *et al.* 2009](#_ENREF_31))  ([Inoue *et al.* 2009](#_ENREF_12))  ([Wang *et al.* 2009](#_ENREF_50)) | | | This study was previously included in the Graphistrength dossier (2015) as Rel 2, but cannot be located anymore.  The dossier also included two other studies (Rel 2 and Rel 4) where test material was not the same but showed no sensitising properties;  Other types of MWCNT; produced by same type production process (CVD), comparable dimensions and purity.  Functionalised MWCNT which may have higher biocompatibility than unfunctionalised;  In sum the results with the different type of MWCNT support the weight of evidence. |  |  |  |
| **Eye Irritation** | | | | **LOW: MWCNT (is slightly irritating to rabbit eyes, not warranting classification)** | | | | |  |  |  |
|  | | Eye Irritation | | Slight irritation of rabbit eyes (redness of conjunctivae) which was reversible within 72h. No classification required.  *In vivo* and *in vitro* (HE-CAM test = Hen's Egg Test-Chorioallantoic Membrane) studies with other types of MWCNT (long and short) suggest slight irritation (reversible conjunctival redness and discharge) not warranting classification or no irritating effect.  In an in vivo study another type of MWCNT (Graphistrength) showed irritating effects (chemosis, redness, iris lesions, corneal opacity) were observed. These were interpreted as secondary to abrasive (mechanical) action as no corneal opacification observed in BCOP (Bovine Corneal Opacity and Permeability) test. | Study report (2006)  ([MWCNT REACH Dossier](#_ENREF_25))  ([Kishore et al. 2009](#_ENREF_15); [Ema et al. 2011](#_ENREF_7))  ([Graphite REACH Dossier](#_ENREF_9) ; [OECD; 2015](#_ENREF_32)) | | | Rel 1; OECD TG 405  Rel 2; HE-CAM test, No TG, (WoE)  Rel 1; OECD TG 405 |  |  |  |
| **Dermal Irritation** | | | | **VERY LOW: MWCNT is not irritating to skin** | | | | |  |  |  |
|  | | Dermal Irritation | | No irritating or corrosive properties in dermal irritation (corrosion) test in vivo and reconstructed human skin (in vitro).  No significant increase of IL-1alpha (marker of irritation) in human reconstructed epidermis in vitro  *In vivo* and *in vitro* (Episkin-SM) studies with other types of MWCNT (long and short) suggest no irritating effect or slight irritation which do not warrant classification. | 3 study reports (2006)  ([MWCNT REACH Dossier](#_ENREF_25))  ([Kishore *et al.* 2009](#_ENREF_15); [Ema *et al.* 2011](#_ENREF_7)) | Rel 1; OECD TG 404  Rel 1; OECD TG 431  Rel 2; | | |  |  |  |
| **Endocrine activity** | | | | **No data available** | | | | |  |  |  |
|  | |  | |  |  |  | | |  |  |  |
| **Immunotoxicity** | | | | **No data available** | | | | |  |  |  |
|  | | Immune System Effects | |  |  |  | | |  |  |  |
| **ECOTOXICITY** | | | | | | | | |  |  |  |
| ECOSAR Class | | | | Not applicable | | | | |  |  |  |
| **Acute Aquatic Toxicity** | | | | **LOW: Effect values from experimental studies for fish and daphnia (biomass) are above the reference values used for hazard designation (LC/EC_50_ values for fish and daphnia > 100 mg/L).** | | | | |  |  |  |
| Fish LC_50_ | | | | *Danio rerio* LC_50_ (48h) >100 mg/L | Study report (2007) ([MWCNT REACH Dossier](#_ENREF_25)) | Rel 1; EU Method C.1; Baytubes C 150 P; | | |  |  |  |
|  |  |  |  | *Danio rerio* LC_50_ (48h) >100 mg/L | Study report (2010) ([MWCNT REACH Dossier](#_ENREF_25)) | Rel 1; OECD Guideline 204; | | |  |  |  |
|  |  |  |  | Studies with other types of MWCNT show comparable results indicating low fish toxicity | ([Graphite REACH Dossier](#_ENREF_9) ; [OECD; 2015](#_ENREF_32)) | (other MWCNT type)  Supports weight of evidence | | |  |  |  |
| Daphnid LC_50_ | | | | *Daphnia magna STRAUSS* EC_50_ (24h) >100 mg/L | Study report (2006) ([MWCNT REACH Dossier](#_ENREF_25)) | Rel 1; EU Method C.1; | | |  |  |  |
|  | | | | *Daphnia magna STRAUSS* EC_50_ (48h) >100 mg/L | Study report (2010) ([MWCNT REACH Dossier](#_ENREF_25)) | Rel 1; OECD Guideline 202; | | |  |  |  |
|  |  |  |  | Studies with other types of MWCNT show comparable results indicating low toxicity for daphnids | ([Graphite REACH Dossier](#_ENREF_9) ; [OECD; 2015](#_ENREF_32)) | (other MWCNT type)  Supports weight of evidence | | |  |  |  |
| Green Algae EC_50_ | | | | *Desmodesmus subspicatus* EC_50_ (72h): 34 mg/L; NOEC (72h) 8.9 mg/L; EC_10_ (72h) 8.9 mg/L; (growth reduction due to reduced light intensity) | Study reports (2007, 2011) ([MWCNT REACH Dossier](#_ENREF_25)) | Rel 1; EU Method C.3; | | |  |  |  |
|  | | | | *Desmodesmus subspicatus* EC50 (72h): 278 mg/L; NOEC (72h) 4.3 mg/L; EC_10_ (72h) 16 mg/L; (growth reduction due to reduced light intensity) | Study reports (2007, 2011) ([MWCNT REACH Dossier](#_ENREF_25)) | Rel 1; EU Method C.3; | | |  |  |  |
| **Chronic Aquatic Toxicity** | | | | **LOW: Effect values from experimental studies for fish and daphnia indicate no effects at the saturation limit.** | | | | |  |  |  |
| Fish ChV | | | | *Danio rerio* EC_10_ (10d) > 100 mg/L | Study report (2010) ([MWCNT REACH Dossier](#_ENREF_25)) | Rel 1; OECD Guideline 212; | | |  |  |  |
| Daphnid ChV | | | | *Daphnia magna* NOEC (21d) > 25mg/L | Study report (2010) ([MWCNT REACH Dossier](#_ENREF_25)) | Rel 1; OECD Guideline 211; | | |  |  |  |
| Green Algae ChV | | | | See above (acute) |  |  | | |  |  |  |
|  | | | | Studies with other types of MWCNT show comparable results indicating low chronic toxicity for fish daphnids and algae | ([Graphite REACH Dossier](#_ENREF_9) ; [OECD; 2015](#_ENREF_32)) | (other MWCNT type)  Supports weight of evidence | | |  |  |  |
| Sediment organisms | | | | No information available  Study with other MWCNT type showed no chronic effects in larvae of chironomidae and terrestrial microorganisms | *(*[*OECD; 2015*](#_ENREF_32)*)* |  | | |  |  |  |
| **ENVIRONMENTAL FATE** | | | | | | | | |  |  |  |
| **Transport** | | | | | | | | |  |  |  |
|  | **Henry's Law constant**  **(atm-m^3^/mole)** | | Not applicable as solid | | ([MWCNT REACH Dossier](#_ENREF_25)) |  | | |  |  |  |
|  | **Sediment/Soil Adsorption/Desorption - K_OC_** | | Study technically not feasible; | | ([MWCNT REACH Dossier](#_ENREF_25)) |  | | |  |  |  |
|  | | **Level III Fugacity Model** | Not applicable as solid | | ([MWCNT REACH Dossier](#_ENREF_21)) |  | | |  |  |  |
| **Persistence** | | ***HIGH*: As inorganic material MWCNT is not expected to biodegrade or oxidise under typical environmental conditions. Minor degradation processes for MWCNT under typical environmental conditions were identified.** | | | | | | |  |  |  |
| Water | | **Ready Biodegradation** | | 0 % after 28 d (O2 consumption)🡪 no biodegradation observed | Study report (2006) ([MWCNT REACH Dossier](#_ENREF_25)) | | | Rel 1; EU Method C.4-D; |  |  |  |
|  | | **Aerobic Biodegradation** | | Biodegradation in water not performed as only organic substances can be degraded by bacteria and MWCNT do not have chemical functions or moieties which might react with water. | expert judgement  ([MWCNT REACH Dossier](#_ENREF_25)) | | |  |  |  |  |
|  |  |  |  | Naturally occurring enzymes or organelles have been shown to degrade CNTs and thereby diminish their potential environmental risks if the nanotubes are exposed to such enzymes in the natural environment. No quantitative data is available | Reviewed in ([Petersen *et al.* 2011](#_ENREF_35)) | | |  |  |  |  |
|  | | **Volitalisation half-life for Model River** | | No information available |  | | |  |  |  |  |
| Soil | | **Aerobic Biodegradation** | | scientifically unjustified; Biodegradation tests are not applicable to inorganic substances as only organic substance can be degraded by bacteria to form carbon dioxide. | expert judgement  ([MWCNT REACH Dossier](#_ENREF_25)) | | |  |  |  |  |
|  |  | **Anaerobic Biodegradation** | | No information available |  | | |  |  |  |  |
|  |  | **Soil Biodegradation with Product Identification** | | No information available |  | | |  |  |  |  |
|  | | **Sediment/Water Biodegradation** | | Biodegradation in water and soil not performed as only organic substances can be degraded by bacteria | expert judgement  ([MWCNT REACH Dossier](#_ENREF_25)) | | |  |  |  |  |
| **Air** | | **Atmospheric Half-life** | | No information available |  | | |  |  |  |  |
| **Reactivity** | | **Photolysis** | | No information available |  | | |  |  |  |  |
|  |  | **Hydrolysis** | | study scientifically unjustified | expert judgement  ([MWCNT REACH Dossier](#_ENREF_25)) | | |  |  |  |  |
|  | |  | |  |  | | |  |  |  |  |
| **Environmental Half-life** | | | | No information available |  | | |  |  |  |  |
| **Bioaccumulation** | | | | ***LOW*: The bioaccumulation potential of MWCNT is estimated low due to its high molecular weight and diameter** | | | | |  |  |  |
|  | | **Fish BCF** | | Study not conducted as MWCNT due to their high molecular weight > 1100 g/mol and a diameter D_max_ average of >1.7 nm have a low potential for bioaccumulation. | expert judgement  ([MWCNT REACH Dossier](#_ENREF_25)) | | |  |  |  |  |
|  | |  | | Significant nanotube accumulation and limited depuration was observed for D. magna across a relatively limited range of experimental conditions. MWCNT detected in oligochaete (earthworms) were associated with sediments remaining in the organism guts and not absorbed into cellular tissues. | Reviewed in ([Petersen *et al.* 2011](#_ENREF_35)) | | | Type of MWCNT not described |  |  |  |

**Part2:**

# Draft GreenScreen^®^ Assessment for Multi-Walled Carbon Nanotubes Nanocyl/Baytubes(EC 936-414-1)

**Method Version: GreenScreen^®^ Version 1.2^^[[3]](#footnote-3)^^**

**Assessment Type^^[[4]](#footnote-4)^^: Non-Verified – Draft for DEROCA project based on data in REACH registration dossier (list No 936-414-1) covering Nanocyl NC7000 and Baytubes C150P^^[[5]](#footnote-5)^^**

**Abstract**

MWCNT (list No 936-414-1) are used for applications in energy storage, automotive parts, boat hulls, sporting goods, water filters, thin-film electronics, coatings, actuators and electromagnetic shields. MWCNT are also used as synergists to improve the performance of non-halogenated flame retardants and to reduce their loading ([NANOCYL S.A. 2012](#_ENREF_29)).

MWCNT (list No 936-414-1) could be assigned a GreenScreen® Benchmark Score of 2 (“Use but Search for Safer Substitutes”) or 3 (Use but Still Opportunity for Improvement). MWCNT has Very High persistence (P) and High Group II Human Toxicity (Inhalation toxicity (AT). This would correspond to GreenScreen® benchmark classification 1c in CPA 2011. However as persistency should not be considered for the fate of inorganic materials, GreenScreen® benchmark classification 2f applies. The score for the repeated inhalation toxicity may be discussed and a score of M may also be valid. This would lead to an overall GreenScreen® Benchmark Score of 3, corresponding to a classification 3c.

Data gaps (DG) exist for endocrine activity (E) and respiratory sensitisation (SnR). Despite the hazard data gaps MWCNT meets the requirements for a GreenScreen® Benchmark Score of 2/3.

**Introduction**

No EPA Alternatives Assessment for MWCNT is available; A draft following the criteria is presented above (part 1).

The GreenScreen assessment is based on the information reported in the REACH registration Dossier (Chemicals Safety Report) for list No 936-414-1, as provided by the registrants and additional information on the same type of MWCNT ([OECD; 2015](#_ENREF_32)). The studies presented in the dossier cover mainly two types of MWCNT, namely Nanocyl NC7000 and Baytubes C150P.

Hazard classification levels reported in the DfE profiles and in this GreenScreen report may differ due to differences between criteria as defined in the DfE "Alternatives Assessment Criteria for Hazard Evaluation ([US-EPA 2011](#_ENREF_47)) and the GreenScreen for Safer chemicals v1.2 methods ([Clean Production Action 2013](#_ENREF_4)). Any differences in interpretation are explained in this GreenScreen draft report.

| **GreenScreen^®^ Assessment Prepared By:** | **GreenScreen^®^ Assessment Quality Control Performed By:** |
| --- | --- |
| Name: Karin Aschberger et al. | Name: |
| Title: Scientific Project Officer | Title: |
| Organization: | Organization: |
| Date: October 2016 | Date: |
| Assessor Type (Licensed GreenScreen Profiler, Authorized GreenScreen Practitioner or Unaccredited): UNACCREDITED |  |

**Confirm application of the *Disclosure and Assessment Rules and Best Practice*^^[[6]](#footnote-6)^^:** (List disclosure threshold and any deviations)

**Chemical Name (CAS #): Multi-Walled Carbon Nanotubes (MWCNT), synthetic graphite in tubular shape (list No 936-414-1)**

**Also Called: Nanocyl NC7000**

**Suitable analogs or moieties of chemicals used in this assessment (CAS #’s): Baytubes C150P**

**Chemical Structure(s): synthetic graphite in tubular shape**

*Note: Include chemical structure(s) of all suitable analogs (and /or moieties) used in the assessment.

**Notes related to production specific attributes^^[[7]](#footnote-7)^^:**

**For Inorganic Chemicals and relevant particulate organics (*if not relevant, list NA*)**

**MWCNT are an inorganic compound. They are synthetic graphite in tubular shape; and consist of >90% carbon.**

**Define Properties:**

1. Particle size **tube diameter d90 ≤ 30nm); length: 0.2** **µm - 5 µm (calculated mean value; 1.5 µm; TEM and SEM)**
2. Structure **amorphous**
3. Mobility **water solubility:** **< 2 mg/L (practically insoluble)**
4. Bioavailability **MWCNT are not absorbed through skin and are estimated to have poor systemic absorption through the lungs and gastrointestinal tract**

**Identify Applications/Functional Uses:**

**1. synergistic flame retardant**

**GreenScreen Benchmark Score and Hazard Summary Table:^^[[8]](#footnote-8)^,^[[9]](#footnote-9)^,^[[10]](#footnote-10)^,^[[11]](#footnote-11)^^ [***Multi-walled carbon nanotubes NC 7000*] is suggested to be assigned a Benchmark Score of 2/3 depending on whether the effects following repeated exposure to low concentrations are considered of HIGH or MODERATE concern. Applying the concentration thresholds it would be high, considering that not classification is proposed, it could be considerate as moderate. The very high persistency is not considered for scoring as MWCNT are inorganic (but not bioaccumulating).

Note: Hazard levels (Very High (vH), High (H), Moderate (M), Low (L), Very Low (vL)) in *italics* reflect estimated values, authoritative B lists, screening lists, weak analogues, and lower confidence. Hazard levels in **BOLD** font are used with good quality data, authoritative A lists, or strong analogues. Group II Human Health endpoints differ from Group II* Human Health endpoints in that they have four hazard scores (i.e., vH, H, M and L) instead of three (i.e., H, M and L), and are based on single exposures instead of repeated exposures.

**Environmental Transformation Products and Ratings^^[[12]](#footnote-12)^^:**

**Identify feasible and relevant environmental transformation products** **(i.e., dissociation products, transformation products, valence states)** **and/or moieties of concern**^^[[13]](#footnote-13)^^

| **Functional Use** | **Life Cycle Stage** | **Transformation Pathway** | **Environmental**  **Transformation Products** | **CAS #** | **Feasible and Relevant?** | **GS List Translator Score or GSBenchmark Score** |
| --- | --- | --- | --- | --- | --- | --- |
|  |  |  | Elemental carbon |  |  |  |

**Introduction**

Multi-walled carbon nanotubes (MWCNT) consist of multiple rolled layers (concentric tubes) of graphene. Depending on the synthesis and purification methods ([Prasek *et al.* 2011](#_ENREF_38)) they may differ in their form (length, diameter) and physico-chemical properties (metal content, e.g. Fe, Co, Ni, aggregation, agglomeration, surface chemistry and functionalisation) which may all have an impact on their toxicological profile ([Donaldson *et al.* 2010](#_ENREF_5); [Braakhuis *et al.* 2014](#_ENREF_2)).

**Hazard Classification Summary Section:**

**Group I Human Health Effects (Group I Human)**

**Carcinogenicity (C) Score (H, M or L):** *L*

*MWCNT LIST No 936-414-1* is suggested to be assigned a score of *LOW* for carcinogenicity based on the absence of tumours in available studies with short, tangled, low-density agglomerate form of MWCNT: Rat intraperitoneal (2 year bioassay): no neoplastic effects up to 20 mg/animal (NC 7000; ([Muller *et al.* 2009](#_ENREF_23)), Rat 90 day inhalation (+6 months postexposure): 0.1 mg/m^3^ NOAEL (Baytubes) ([Pauluhn 2010a](#_ENREF_33)); Rat 90 day inhalation LOAEL (NC 7000) ([Ma-Hock *et al.* 2009](#_ENREF_19)); Both types induced granulomatous inflammation;

No oral chronic study available.

*MWCNT list No 936-414-1* is not GHS classified and there are currently no proposals/notifications to classify it for carcinogenicity. There are no structural alerts for carcinogenicity. MWCNT registered under REACH are included in the Community Rolling Action Plan (CORAP) ^[[14]](#footnote-14)^ for substance evaluation under REACH due to the following reasons: suspected C (Carcinogen), wide dispersive use, exposure of environment, consumer use, cumulative exposure, exposure of workers, environmental fate and ecotoxicity.

IARC: classified *MWCNT list No 936-414-1*  as cat. 3 "Not classifiable as to its carcinogenicity to humans". Note, that there are no Greenscreen^®^ criteria for IARC category 3;

**Mutagenicity/Genotoxicity (M) Score (H, M or L):** *L*

*MWCNT list No 936-414-1* is suggested to be assigned a score of *LOW* for mutagenicity based on the absence of positive effects in most *in vitro* studies. One *in vivo* study showing slightly positive effects was considered not reliable by the registrant; other *in vivo* studies were negative.

Negative in Ames test up to 5000 μg/plate (w/o S9 mix) (Baytubes C150P; ([Wirnitzer *et al.* 2009](#_ENREF_51)); study report 2010 ([MWCNT REACH Dossier](#_ENREF_25)).

Negative for Chinese Hamster lung fibroblasts V79 p to 100 μg/ml (w/o S9 mix) study report 2010 ([MWCNT REACH Dossier](#_ENREF_25)).

Negative for Chinese Hamster lung fibroblasts V79, (Baytubes C150P; ([Wirnitzer *et al.* 2009](#_ENREF_51)); study reports 2006, 2007 ([MWCNT REACH Dossier](#_ENREF_25)); Inconsistent results in RLE cells (NC 7000; ([Muller *et al.* 2008a](#_ENREF_21); [Muller *et al.* 2008b](#_ENREF_22)).

*MWCNT LIST No 936-414-1* is not GHS classified and there are currently no proposals to classify it for mutagenicity. There are no structural alerts for mutagenicity.

**Reproductive Toxicity (R) Score (H, M, or L):** *L*

*MWCNT LIST No 936-414-1* is suggested to be assigned a score of *LOW* for reproductive toxicity based on expert judgment ([MWCNT REACH Dossier](#_ENREF_25)): lack of systemic availability; no effects in female and male reproductive organs in subchronic inhalation studies up to concentrations that caused lung toxicity.

*MWCNT LIST No 936-414-1* is not GHS classified and there are currently no proposals to classify it for reproductive toxicity.

**Developmental Toxicity incl. Developmental Neurotoxicity (D) Score (H, M or L):** *L*

*MWCNT LIST No 936-414-1* is suggested to be assigned a score of *LOW* for developmental toxicity based on expert judgment ([MWCNT REACH Dossier](#_ENREF_25)): lack of systemic availability; no effects in female and male reproductive organs in subchronic inhalation studies up to concentrations that caused lung toxicity (Registration dossier)

*MWCNT LIST No 936-414-1* is not GHS classified and there are currently no proposals to classify it for developmental toxicity.

**Endocrine Activity (E) Score (H, M or L):** *L*

*MWCNT LIST No 936-414-1* is suggested to be assigned a score of *LOW* for endocrine activity based on expert judgment as based on the structure and chemical composition MWCNT are unlikely to have endocrine activity.

**Group II and II* Human Health Effects (Group II and II* Human)**

*Note: Group II and Group II* endpoints are distinguished in the v 1.2 Benchmark system (the asterisk indicates repeated exposure). For Systemic Toxicity and Neurotoxicity, Group II and II* are considered sub-endpoints. When classifying hazard for Systemic Toxicity/Organ Effects and Neurotoxicity endpoints, repeated exposure results are required and preferred. Lacking repeated exposure results in a data gap. Lacking single exposure data does not result in a data gap when repeated exposure data are present (shade out the cell in the hazard table and make a note). If data are available for both single and repeated exposures, then the more conservative value is used.*

**Acute Mammalian Toxicity (AT) Group II Score (vH, H, M or L):** *M*

*MWCNT LIST No 936-414-1* is suggested to be assigned a score of *MODERATE* for acute mammalian toxicity. An LC_50_ (6h)> 241 mg/m^3^ (> 0.241 mg/L) air carried out with Baytubes C150P ([Ellinger-Ziegelbauer and Pauluhn 2009](#_ENREF_6)) was determined in a rat inhalation study. This concentrations is below the threshold for VERY HIGH (LC_50_ ≤ 0.5 mg/L). However, it should be noted that 0.241 mg/L MWCNT in that study was the maximum technically attainable concentration and no deaths occurred at that concentration The animals showed transient clinical signs (irregular and laboured breathing patterns up to postexposure day 2 and reduced body weights); Irreversible pulmonary inflammatory response (influx of polymorphonuclear cells) was observed at all reading points (7, 28, 90 days after exposure with peak concentrations shortly after exposure (day 7).

Greenscreen® has no criteria for acute NOAECs and therefore it is difficult to apply them to (nano)particles which cannot be tested up to the threshold concentrations of the different scores.

*MWCNT LIST No 936-414-1* is not GHS classified and there are currently no proposals (by registrant) to classify it for acute inhalation toxicity; considering this, the score would be LOW. However as there are suggestive animal studies showing irreversible effects at low concentrations following inhalation "no basis for concern identified" is not supported. Consequently a score of MODERATE is suggested for inhalation toxicity, considering that high concentrations inducing severe acute effects are very unlikely to occur.

Oral and dermal toxicity are considered LOW with LD_50_ of > 2000 mg/kg bw for oral (study reports 2006, 2008 ([MWCNT REACH Dossier](#_ENREF_25))) and dermal exposure (study report 2006 ([MWCNT REACH Dossier](#_ENREF_25))).

**Systemic Toxicity/Organ Effects incl. Immunotoxicity (ST)**

**(ST-single) Group II Score (single dose: vH, H, M or L);** *M*

*MWCNT LIST No 936-414-1* is suggested to be assigned a score of *MODERATE* for systemic toxicity/organ effects based on single exposure. No severe effects were observed up to the maximum technically attainable concentration in the acute inhalation study with the similar MWCNT type Baytubes C150P ([Ellinger-Ziegelbauer and Pauluhn 2009](#_ENREF_6)). Observed effects were transient clinical signs (irregular and laboured breathing patterns up to postexposure day 2 and reduced body weights) and irreversible pulmonary inflammatory response. Discoloration of the lungs and enlargement/discoloration of lung-associated lymph nodes were apparent at all sacrifices at 241 mg/m^3^. Enlarged and/or foamy macrophages with dark cytoplasmatic spots were observed in both concentration groups (11 and 241 mg/m^3^). At 241 mg/m^3^ bronchiolo-alveolare hypercellularity, focal septal thickening and focal increased septal collagen with no time-dependent differences (28 or 90 day) were observed.

If these effects are considered local effects, no GHS classification for STOT SE is required^^[[15]](#footnote-15)^^. In case the effects on the lung-associated lymph nodes would be considered as relevant/severe systemic effects (on the immune system), a classification as STOT-SE Category 1 based on the low concentrations at which the effects occurred, could be proposed. Such classification would trigger a score of VERY HIGH.

Based on the low (or absent?) systemic availability of MWCNT following inhalation exposure and low toxicity observed following oral and dermal exposure, such a stringent classification is not supported here. In addition, as the acute inhalation study included a post-exposure period up to 90 days it can be assumed that (other) severe systemic toxicity effects following single exposure may have been detected. Such effects were not reported.

However as there are suggestive animal studies showing irreversible effects at low concentrations following inhalation it cannot be supported that there is "no basis for concern identified", a score of *MODERATE* is suggested for inhalation toxicity.

**(ST-repeat) Group II* Score (repeated dose: H, M, L):** *H*

*MWCNT LIST No 936-414-1* is suggested to be assigned a score of *HIGH* for systemic toxicity/organ effects based on repeated exposure.

Overload associated inflammation was observed at the higher exposure concentrations (1.5 and 6 mg/m^3^) associated with sustained pulmonary inflammation ([Pauluhn 2010a](#_ENREF_33)). Granulomas and alveolar hyperplasia were observed ≥ 6 mg/m^3^. Effects were not reversible within the post-exposure period of 6 months (≈0.5 t_1/2_); the retention time for 6 mg/m^3^ was calculated to be 375 d. Translocation of MWCNT into lung associated lymph nodes was detectable only after 13 weeks and sustained elevations in neutrophylic granulocytes in the bronchoalveolar lavage occurred at the two highest concentrations with borderline effects at 0.4 mg/m^3^. Apart from Lymph node associated nods (LALNs) no exposure concentration extrapulmonary systemic toxicity was observed (Baytubes; ([Pauluhn 2010b](#_ENREF_34); [Pauluhn 2010a](#_ENREF_33)). A second subchronic inhalation study confirms the absence of any pathological response in major organs such as the liver, kidney or heart and confirms in principle the findings of adverse pulmonary effects (Nanocyl NC 7000; ([Ma-Hock *et al.* 2009](#_ENREF_19))).

The lowest exposure level tested, 0.1 mg/m^3^, was a NOAEC for Baytubes and a LOAEC for Nanocyl NC7000 (minimal granulomatous-type inflammation in the lungs and lung-associated lymph nodes).

Depending on the interpretation of the criteria, the score could be MODERATE to HIGH.

Strictly applying the threshold values a NOAEC/LOAEC of 0.0001 mg/L leads to a score of *HIGH*. Considering the criteria for a core of HIGH would require a GHS Category 1 classification or evidence of adverse effects in humans or a weight of evidence demonstrating potential for adverse effects in humans, a score of *MODERATE* based on suggestive animal studies could also be proposed in this assessment (see below discussion on the benchmark score).

**Neurotoxicity (N)**

**(N-single) Group II Score (single dose: vH, H, M or L):** *L*

*MWCNT LIST No 936-414-1* is suggested to be assigned a score of *LOW* for neurotoxicity based on expert judgment: lack of systemic availability; no effects were reported for animals following single inhalation exposure and up to 90 days post-exposure period ([Pauluhn 2010a](#_ENREF_33)).

*MWCNT LIST No 936-414-1* is not GHS classified and there are currently no proposals (by the registrant) to classify it for neurotoxic effects.

**(N-repeat) Group II* Score (repeated dose: H, M, L):** *L*

*MWCNT LIST No 936-414-1* is suggested to be assigned a score of *LOW* for neurotoxicity based on expert judgment: lack of systemic availability; no effects were reported for animals following repeated subchronic exposure and up to 6 months post-exposure period.

*MWCNT LIST No 936-414-1* is not GHS classified and there are currently no proposals (by the registrant) to classify it for neurotoxic effects.

**Skin Sensitization (SnS) Group II* Score (H, M or L): L**

*MWCNT LIST No 936-414-1* is suggested to be assigned a score of *LOW* for skin sensitization based on negative results (Baytubes C150 P) in a guinea pigs maximisation test (study report (2007) ([MWCNT REACH Dossier](#_ENREF_25))).

**Respiratory Sensitization (SnR) Group II* Score (H, M or L):** *L*

*MWCNT LIST No 936-414-1* is suggested to be assigned a score of *LOW* based on expert judgement. Studies carried out with other types of MWCNT did not show sensitizing properties ([Ronzani *et al.* 2014](#_ENREF_40)). No indication for respiratory sensitization were reported in two subchronic inhalation studies with MWCNT list No 936-141-1 ([Ma-Hock *et al.* 2009](#_ENREF_19); [Pauluhn 2010a](#_ENREF_33)).

**Skin Irritation/Corrosivity (IrS) Group II Score (vH, H, M or L): L**

*MWCNT LIST No 936-414-1* is suggested to be assigned a score of **LOW** for skin irritation/corrosivity based on the absence of irritating effects in 3 study reports (2006; ([MWCNT REACH Dossier](#_ENREF_25))).

**Eye Irritation/Corrosivity (IrE) Group II Score (vH, H, M or L): L**

*MWCNT LIST No 936-414-1* is suggested to be assigned a score of **LOW** for eye irritation/corrosivity based on results from an in vivo eye irritation test (study report 2006; ([MWCNT REACH Dossier](#_ENREF_25)). Slight (redness of conjunctivae)/reversible (within 72h) irritation on rabbit eyes was observed, which does not warrant GHS classification.

**Ecotoxicity (Ecotox)**

**Acute Aquatic Toxicity (AA) Score (vH, H, M or L): L**

*MWCNT LIST No 936-414-1* is suggested to be assigned a score of LOW for acute aquatic toxicity as the effect values from experimental studies for fish, daphnia and algae with Baytubes C150P and Nanocyl NC7000 indicate that there are no effects observed up to the saturation limit (study reports 2006, 2007, 2010, 2011, ([MWCNT REACH Dossier](#_ENREF_25)).

**Chronic Aquatic Toxicity (CA) Score (vH, H, M or L): L**

*MWCNT LIST No 936-414-1* is suggested to be assigned a score of LOW for chronic aquatic toxicity as effect values from experimental studies for fish and daphnia indicate no effects at the saturation limit (study reports 2010, ([MWCNT REACH Dossier](#_ENREF_25))). EC10 values for fish are 100 mg/l, NOEC for daphnia: >25 mg/L, NOECs for algae: 8.9 and 4.3 mg/L. The values for fish and daphnia are above the threshold for MODERATE score (NOAEC/LOEC: 1-10 mg/L), whereas those for algae are slightly below. No GHS classification was requested by the registrant.

**Environmental Fate (Fate)**

**Persistence (P) Score (vH, H, M, L, or vL):** *vH*

*MWCNT LIST No 936-414-1* is suggested to be assigned a score of *VERY HIGH* for persistence. As inorganic material MWCNT is not expected to biodegrade or oxidise under typical environmental conditions. MWCNT showed no (ready) biodegradation after 28d (study report 2006, ([MWCNT REACH Dossier](#_ENREF_25))). Other biodegrdation tests in water and soil were waived as scientifically not justified. Minor degradation processes for MWCNT under typical environmental conditions were identified([Petersen *et al.* 2011](#_ENREF_35)).

**Bioaccumulation (B) Score (vH, H, M, L, or vL):** *L*

*MWCNT LIST No 936-414-1* is suggested to be assigned a score of *LOW* for bioaccumulation based on expert judgement

MWCNT due to its high molecular weight > 1100 g/mol and a diameter Dmax average of >1.7 nm have a low potential for bioaccumulation. Few studies describe accumulation and limited depuration in daphnia magna and no absorption in oligochaete ([Petersen *et al.* 2011](#_ENREF_35)).

**Physical Hazards (Physical)**

**Reactivity (Rx) Score (vH, H, M or L):** *L*

*MWCNT LIST No 936-414-1* is suggested to be assigned a score of *LOW* for reactivity. MWCNT did not show exothermic reactions up to 450°C (study report 2006, ([MWCNT REACH Dossier](#_ENREF_25))). Expert judgement suggests that from its chemical composition, an explosion hazard can be excluded (study report 2007, ([MWCNT REACH Dossier](#_ENREF_25))). MWCNT has no chemical groups associated with oxidising behaviour. No GHS classification is suggested by the registrant.

**Flammability (F) Score (vH, H, M or L): L**

*MWCNT LIST No 936-414-1* is suggested to be assigned a score of L for flammability. They are not highly flammable, did not liberate flammable gases in contact with water had no pyrogenic properties (study report 2007, ([MWCNT REACH Dossier](#_ENREF_25))). They also showed brief ignition and rapid extinction in burning behaviour test (study report 2006, ([MWCNT REACH Dossier](#_ENREF_25))). No GHS classification is suggested by the registrant.

**Applying the Benchmark:**

**Benchmark Criteria Worksheet**

MWCNT considering high inhalation toxicity 🡪 leading to Benchmark 2

| **Benchmark** | **a** | **b** | **c** | **d** | **e** | **f** | **g** |
| --- | --- | --- | --- | --- | --- | --- | --- |
| **1** | no | no | no* | no | no |  |  |
| **2** | no | no | no* | no | no | Yes ** | no |
| **3** | yes | no | yes | no |  |  |  |
| **4** | no |  |  |  |  |  |  |

* persistency not considered as inorganic material;

**high inhalation toxicity

MWCNT considering moderate inhalation toxicity 🡪 leading to Benchmark 3

| **Benchmark** | **a** | **b** | **c** | **d** | **e** | **f** | **g** |
| --- | --- | --- | --- | --- | --- | --- | --- |
| **1** | no | no | no | no | no |  |  |
| **2** | no | no | no* | no | no | no | no |
| **3** | no | no | Yes** | no |  |  |  |
| **4** | no |  |  |  |  |  |  |

* persistency not considered as inorganic material;

**moderate inhalation toxicity

**Discussion:**

Benchmark 1: As MWCNT are inorganic, persistency is not considered in combination with group II Human Hazard. Therefore MWCNT does not meet the criteria for Benchmark 1(c). Scores for Group I Hazard (CMR) are considered LOW.

Benchmark 2: if inhalation toxicity of MWCNT is considered HIGH, then it meets the criteria of Benchmark 2(f). As inorganic material the criteria of 2(c) do not apply.

Benchmark 3: if inhalation toxicity of MWCNT was considered of MODERATE concern it would meet the criteria of Benchmark 3(c).

**Gap analysis:**

For several endpoints no data was presented, though with justifications given.

MWCNT passes the data gap analysis for Benchmark 2 and 3 if the justification for not providing reproductive or developmental toxicity or excluding concern for such endpoints without testing, is accepted. Data for all other endpoints are provided, except some of permissible data gaps, e.g. endocrine activity.

**Hazard Benchmark Acronyms** (alphabetical order)

(AA) Acute Aquatic Toxicity

(AT) Acute Mammalian Toxicity

(B) Bioaccumulation

(C) Carcinogenicity

(CA) Chronic Aquatic Toxicity

(Cr) Corrosion/ Irritation (Skin/ Eye)

(D) Developmental Toxicity

(E) Endocrine Activity

(F) Flammability

(IrE) Eye Irritation/Corrosivity

(IrS) Skin Irritation/Corrosivity

(M) Mutagenicity and Genotoxicity

(N) Neurotoxicity

(P) Persistence

(R) Reproductive Toxicity

(Rx) Reactivity

(SnS) Sensitization- Skin

(SnR) Sensitization- Respiratory

(ST) Systemic/Organ Toxicity

# References

Binderup, M.-L., Bredsdorff, L., Beltoft, V. M., Mortensen, A., Löschner, K., Larsen, E. H. and Eriksen, F. D. (2013). Systemic Absorption of Nanomaterials by Oral Exposure; Part of the ”Better control of nano” initiative 2012-2015. Copenhagen; from <http://orbit.dtu.dk/files/59606121/Systemic%20absorption%20of%20nanomaterials%20by%20oral%20exposure%20978-87-93026-51-3.pdf>.

Braakhuis, H. M., Park, M. V., Gosens, I., De Jong, W. H. and Cassee, F. R. (2014). "Physicochemical characteristics of nanomaterials that affect pulmonary inflammation." *Particle and Fibre Toxicology* **11**(1): 1-25.

Cao, Y., Jacobsen, N. R., Danielsen, P. H., Lenz, A. G., Stoeger, T., Loft, S., Wallin, H., Roursgaard, M., Mikkelsen, L. and Møller, P. (2014). "Vascular Effects of Multiwalled Carbon Nanotubes in Dyslipidemic ApoE −/− Mice and Cultured Endothelial Cells." *Toxicological Sciences* **138**(1): 104-116.

Clean Production Action (2013). GREENSCREEN® FOR SAFER CHEMICALS - Chemical Hazard Assessment Procedure. Somerville, MA, Clean Production Action, from <http://www.greenscreenchemicals.org/method/full-greenscreen-method>.

Donaldson, K., Murphy, F. A., Duffin, R. and Poland, C. A. (2010). "Asbestos, carbon nanotubes and the pleural mesothelium: a review of the hypothesis regarding the role of long fibre retention in the parietal pleura, inflammation and mesothelioma." *Part Fibre Toxicol* **7**.

Ellinger-Ziegelbauer, H. and Pauluhn, J. (2009). "Pulmonary toxicity of multi-walled carbon nanotubes (Baytubes®) relative to α-quartz following a single 6 h inhalation exposure of rats and a 3 months post-exposure period." *Toxicology* **266**(1–3): 16-29.

Ema, M., Matsuda, A., Kobayashi, N., Naya, M. and Nakanishi, J. (2011). "Evaluation of dermal and eye irritation and skin sensitization due to carbon nanotubes." *Regulatory Toxicology and Pharmacology* **61**(3): 276-281.

Ema, M., Hougaard, K. S., Kishimoto, A. and Honda, K. (2016). "Reproductive and developmental toxicity of carbon-based nanomaterials: A literature review." *Nanotoxicology* **10**(4): 391-412.

Graphite REACH Dossier. "REACH registration dossier: synthetic graphite (EC number: 231-955-3; CAS number: 7782-42-5)." from <http://echa.europa.eu/registration-dossier/-/registered-dossier/16080/1>.

Grosse, Y., Loomis, D., Guyton, K. Z., Lauby-Secretan, B., El Ghissassi, F., Bouvard, V., Benbrahim-Tallaa, L., Guha, N., Scoccianti, C., Mattock, H. and Straif, K. (2014). "Carcinogenicity of fluoro-edenite, silicon carbide fibres and whiskers, and carbon nanotubes." *The Lancet Oncology* **15**(13): 1427-1428.

Hougaard, K. S., Jackson, P., Kyjovska, Z. O., Birkedal, R. K., De Temmerman, P.-J., Brunelli, A., Verleysen, E., Madsen, A. M., Saber, A. T., Pojana, G., Mast, J., Marcomini, A., Jensen, K. A., Wallin, H., Szarek, J., Mortensen, A. and Vogel, U. (2013). "Effects of lung exposure to carbon nanotubes on female fertility and pregnancy. A study in mice." *Reproductive Toxicology* **41**: 86-97.

Inoue, K.-i., Koike, E., Yanagisawa, R., Hirano, S., Nishikawa, M. and Takano, H. (2009). "Effects of multi-walled carbon nanotubes on a murine allergic airway inflammation model." *Toxicology and Applied Pharmacology* **237**(3): 306-316.

Jacobsen, N. R., Wallin, H., De Jong, W., Oomen, A., Brandon, E., Krystek, P., Apostolova, M., Karadjova, I., Cubadda, F., Aureli, F., Maranghi, F., Dive, V., Taran, F. and Czarny, B. (2013). Towards a method for detecting the potential genotoxicity of nanomaterials; Deliverable 7: Identification of target organs and biodistribution including ADME parameters. 3720 BA Bilthoven, THE NETHERLANDSfrom <http://www.nanogenotox.eu/files/PDF/DELIVRABLES2/deliverable7__biodistribution.pdf>.

JRC-Repository; (2014). Multi-Walled Carbon Nanotubes, NM-400, NM-401, NM-402, NM-403: Characterisation and Physico-Chemical Properties; from <http://ihcp.jrc.ec.europa.eu/our_databases/web-platform-on-nanomaterials>.

Kishore, A. S., Surekha, P. and Murthy, P. B. (2009). "Assessment of the dermal and ocular irritation potential of multi-walled carbon nanotubes by using in vitro and in vivo methods." *Toxicology Letters* **191**(2–3): 268-274.

Klimisch, H. J., Andreae, M. and Tillmann, U. (1997). "A Systematic Approach for Evaluating the Quality of Experimental Toxicological and Ecotoxicological Data." *Regulatory Toxicology and Pharmacology* **25**(1): 1-5.

Lim, J.-H., Kim, S.-H., Lee, I.-C., Moon, C., Kim, S.-H., Shin, D.-H., Kim, H.-C. and Kim, J.-C. (2011a). "Evaluation of Maternal Toxicity in Rats Exposed to Multi-Wall Carbon Nanotubes during Pregnancy." *Environmental Health and Toxicology* **26**: e2011006.

Lim, J.-H., Kim, S.-H., Shin, I.-S., Park, N.-H., Moon, C., Kang, S.-S., Kim, S.-H., Park, S.-C. and Kim, J.-C. (2011b). "Maternal exposure to multi-wall carbon nanotubes does not induce embryo–fetal developmental toxicity in rats." *Birth Defects Research Part B: Developmental and Reproductive Toxicology* **92**(1): 69-76.

Ma-Hock, L., Treumann, S., Strauss, V., Brill, S., Luizi, F., Mertler, M., Wiench, K., Gamer, A. O., van Ravenzwaay, B. and Landsiedel, R. (2009). "Inhalation Toxicity of Multiwall Carbon Nanotubes in Rats Exposed for 3 Months." *Toxicological Sciences* **112**(2): 468-481.

Muller, J., Huaux, F., Moreau, N., Misson, P., Heilier, J. F., Delos, M., Arras, M., Fonseca, A., Nagy, J. B. and Lison, D. (2005). "Respiratory toxicity of multi-wall carbon nanotubes." *Toxicology and applied pharmacology* **207**.

Muller, J., Decordier, I., Hoet, P. H., Lombaert, N., Thomassen, L., Huaux, F., Lison, D. and Kirsch-Volders, M. (2008a). "Clastogenic and aneugenic effects of multi-wall carbon nanotubes in epithelial cells." *Carcinogenesis* **29**(2): 427-433.

Muller, J., Huaux, F., Fonseca, A., Nagy, J. B., Moreau, N., Delos, M., Raymundo-Pinero, E., Beguin, F., Kirsch-Volders, M. and Fenoglio, I. (2008b). "Structural defects play a major role in the acute lung toxicity of multiwall carbon nanotubes: toxicological aspects." *Chem Res Toxicol* **21**.

Muller, J., Delos, M., Panin, N., Rabolli, V., Huaux, F. and Lison, D. (2009). "Absence of Carcinogenic Response to Multiwall Carbon Nanotubes in a 2-Year Bioassay in the Peritoneal Cavity of the Rat." *Toxicological Sciences* **110**(2): 442-448.

MWCNT Dossier (2016). "REACH registration dossier: Multi-Walled Carbon Nanotubes (MWCNT), synthetic graphite in tubular shape (List No 936-414-1)." from <http://echa.europa.eu/registration-dossier/-/registered-dossier/13454/7/2/2/?documentUUID=d042b6e7-1608-420a-b644-475a6450fe43>.

MWCNT REACH Dossier. "REACH registration dossier: Multi-Walled Carbon Nanotubes (MWCNT), synthetic graphite in tubular shape (EC number: 936-414-1)." from <http://echa.europa.eu/registration-dossier/-/registered-dossier/13454/7/2/2/?documentUUID=d042b6e7-1608-420a-b644-475a6450fe43>.

Nagai, H., Okazaki, Y., Chew, S. H., Misawa, N., Yamashita, Y., Akatsuka, S., Ishihara, T., Yamashita, K., Yoshikawa, Y. and Yasui, H. (2011). "Diameter and rigidity of multiwalled carbon nanotubes are critical factors in mesothelial injury and carcinogenesis." *Proc Natl Acad Sci U S A* **108**.

Nagai, H., Okazaki, Y., Chew, S. H., Misawa, N., Miyata, Y., Shinohara, H. and Toyokuni, S. (2013). "Intraperitoneal administration of tangled multiwalled carbon nanotubes of 15 nm in diameter does not induce mesothelial carcinogenesis in rats." *Pathology International* **63**(9): 457-462.

Nanocyl (2016). "Technical Data Sheet: NC7000™ Multiwall Carbon Nanotubes." Retrieved 18/04/2016, 2016, from <http://www.nanocyl.com/wp-content/uploads/2016/02/Technical-Data-Sheet-NC7000-V07.pdf>.

NANOCYL S.A. (2012). DEROCA - Development of safer and more Eco-friendly flame Retardant materials based on CNT cO-additives for Commodity Applications

Nanogenotox (2013). Facilitating the safety evaluation of manufactured nanomaterials by characterising their potential genotoxic hazard; from <http://www.nanogenotox.eu/files/PDF/nanogenotox_web.pdf>.

Nygaard, U. C., Hansen, J. S., Samuelsen, M., Alberg, T., Marioara, C. D. and Løvik, M. (2009). "Single-Walled and Multi-Walled Carbon Nanotubes Promote Allergic Immune Responses in Mice." *Toxicological Sciences* **109**(1): 113-123.

OECD; (2015). Dossier MWCNTfrom [www.oecd.org/](http://www.oecd.org/).

Pauluhn, J. (2010a). "Subchronic 13-week inhalation exposure of rats to multiwalled carbon nanotubes: toxic effects are determined by density of agglomerate structures, not fibrillar structures." *Toxicol Sci* **113**.

Pauluhn, J. (2010b). "Multi-walled carbon nanotubes (Baytubes®): Approach for derivation of occupational exposure limit." *Regulatory Toxicology and Pharmacology* **57**(1): 78-89.

Petersen, E. J., Zhang, L., Mattison, N. T., O’Carroll, D. M., Whelton, A. J., Uddin, N., Nguyen, T., Huang, Q., Henry, T. B., Holbrook, R. D. and Chen, K. L. (2011). "Potential Release Pathways, Environmental Fate, And Ecological Risks of Carbon Nanotubes." *Environmental Science & Technology* **45**(23): 9837-9856.

Pothmann, D., Simar, S., Schuler, D., Dony, E., Gaering, S., Le Net, J.-L., Okazaki, Y., Chabagno, J. M., Bessibes, C., Beausoleil, J., Nesslany, F. and Régnier, J.-F. (2015). "Lung inflammation and lack of genotoxicity in the comet and micronucleus assays of industrial multiwalled carbon nanotubes Graphistrength(©) C100 after a 90-day nose-only inhalation exposure of rats." *Particle and Fibre Toxicology* **12**: 21.

Poulsen, S. S., Saber, A. T., Williams, A., Andersen, O., Kobler, C., Atluri, R., Pozzebon, M. E., Mucelli, S. P., Simion, M., Rickerby, D., Mortensen, A., Jackson, P., Kyjovska, Z. O., Molhave, K., Jacobsen, N. R., Jensen, K. A., Yauk, C. L., Wallin, H., Halappanavar, S. and Vogel, U. (2015). "MWCNTs of different physicochemical properties cause similar inflammatory responses, but differences in transcriptional and histological markers of fibrosis in mouse lungs." *Toxicology and applied pharmacology* **284**.

Prasek, J., Drbohlavova, J., Chomoucka, J., Hubalek, J., Jasek, O., Adam, V. and Kizek, R. (2011). "Methods for carbon nanotubes synthesis-review." *Journal of Materials Chemistry* **21**(40): 15872-15884.

Rittinghausen, S., Hackbarth, A., Creutzenberg, O., Ernst, H., Heinrich, U., Leonhardt, A. and Schaudien, D. (2014). "The carcinogenic effect of various multi-walled carbon nanotubes (MWCNTs) after intraperitoneal injection in rats." *Part Fibre Toxicol* **11**.

Ronzani, C., Casset, A. and Pons, F. (2014). "Exposure to multi-walled carbon nanotubes results in aggravation of airway inflammation and remodeling and in increased production of epithelium-derived innate cytokines in a mouse model of asthma." *Archives of Toxicology* **88**(2): 489-499.

Sakamoto, Y., Nakae, D., Fukumori, N., Tayama, K., Maekawa, A., Imai, K., Hirose, A., Nishimura, T., Ohashi, N. and Ogata, A. (2009). "Induction of mesothelioma by a single intrascrotal administration of multi-wall carbon nanotube in intact male Fischer 344 rats." *The Journal of Toxicological Sciences* **34**(1): 65-76.

Sargent, L. M., Porter, D. W., Staska, L. M., Hubbs, A. F., Lowry, D. T., Battelli, L., Siegrist, K. J., Kashon, M. L., Mercer, R. R., Bauer, A. K., Chen, B. T., Salisbury, J. L., Frazer, D., McKinney, W., Andrew, M., Tsuruoka, S., Endo, M., Fluharty, K. L., Castranova, V. and Reynolds, S. H. (2014). "Promotion of lung adenocarcinoma following inhalation exposure to multi-walled carbon nanotubes." *Particle and Fibre Toxicology* **11**: 3-3.

Suzui, M., Futakuchi, M., Fukamachi, K., Numano, T., Abdelgied, M., Takahashi, S., Ohnishi, M., Omori, T., Tsuruoka, S., Hirose, A., Kanno, J., Sakamoto, Y., Alexander, D. B., Alexander, W. T., Jiegou, X. and Tsuda, H. (2016). "Multiwalled carbon nanotubes intratracheally instilled into the rat lung induce development of pleural malignant mesothelioma and lung tumors." *Cancer Science*: n/a-n/a.

Takagi, A., Hirose, A., Nishimura, T., Fukumori, N., Ogata, A., Ohashi, N., Kitajima, S. and Kanno, J. (2008). "Induction of mesothelioma in p53+/ mouse by intraperitoneal application of multi-wall carbon nanotube." *J Toxicol Sci* **33**: 105 - 116.

Takagi, A., Hirose, A., Futakuchi, M., Tsuda, H. and Kanno, J. (2012). "Dose-dependent mesothelioma induction by intraperitoneal administration of multi-wall carbon nanotubes in p53 heterozygous mice." *Cancer Science* **103**(8): 1440-1444.

Thurnherr, T., Brandenberger, C., Fischer, K., Diener, L., Manser, P., Maeder-Althaus, X., Kaiser, J.-P., Krug, H. F., Rothen-Rutishauser, B. and Wick, P. (2011). "A comparison of acute and long-term effects of industrial multiwalled carbon nanotubes on human lung and immune cells in vitro." *Toxicology Letters* **200**(3): 176-186.

US-EPA (2011). Design for the Environment Program Alternatives Assessment Criteria for Hazard Evaluation. Washington; from <http://www2.epa.gov/sites/production/files/2014-01/documents/aa_criteria_v2.pdf>.

Vales, G., Rubio, L. and Marcos, R. (2016). "Genotoxic and cell-transformation effects of multi-walled carbon nanotubes (MWCNT) following in vitro sub-chronic exposures." *Journal of hazardous materials* **306**: 193-202.

Vankoningsloo, S., Piret, J.-P., Saout, C., Noel, F., Mejia, J., Zouboulis, C. C., Delhalle, J., Lucas, S. and Toussaint, O. (2010). "Cytotoxicity of multi-walled carbon nanotubes in three skin cellular models: Effects of sonication, dispersive agents and corneous layer of reconstructed epidermis." *Nanotoxicology* **4**(1): 84-97.

Wang, J., Sun, R. H., Zhang, N., Nie, H., Liu, J. H., Wang, J. N., Wang, H. and Liu, Y. (2009). "Multi-walled carbon nanotubes do not impair immune functions of dendritic cells." *Carbon* **47**(7): 1752-1760.

Wirnitzer, U., Herbold, B., Voetz, M. and Ragot, J. (2009). "Studies on the in vitro genotoxicity of baytubes®, agglomerates of engineered multi-walled carbon-nanotubes (MWCNT)." *Toxicology Letters* **186**(3): 160-165.

1. *This document presents the opinion of the authors and should not be considered as an official document of the European Commission-Joint Research Centre* [↑](#footnote-ref-1)
2. http://echa.europa.eu/documents/10162/dd1871fd-5cbf-4dd8-98dc-90ac4bd62017 [↑](#footnote-ref-2)
3. Use GreenScreen® Assessment Procedure (Guidance) V1.2 [↑](#footnote-ref-3)
4. GreenScreen reports are either “UNACCREDITED” (by unaccredited person), “AUTHORIZED” (by Authorized GreenScreen Practitioner), “CERTIFIED” (by Licensed GreenScreen Profiler or equivalent) or “CERTIFIED WITH VERIFICATION” (Certified or Authorized assessment that has passed GreenScreen Verification Program) [↑](#footnote-ref-4)
5. This draft assessment following the Greenscreen® template is based on information provided for the two MWCNT types Nanocyl NC7000, Baytubes C150P and thus only valid for this MWCNT type; conclusions cannot be extrapolated to any other CNT type. This "assessment" must not be understood as certified GreenScreen® Assessment and should not be used for any other purpose than as case study for the DEROCA project (Grant Agreement n° 308391) (NANOCYL S.A. 2012). [↑](#footnote-ref-5)
6. See GreenScreen Guidance V1.2 [↑](#footnote-ref-6)
7. Note any composition or hazard attributes of the chemical product relevant to how it is manufactured. For example, certain synthetic pathways or processes result in typical contaminants, by-products or transformation products. Explain any differences between the manufactured chemical product and the GreenScreen assessment of the generic chemical by CAS #. [↑](#footnote-ref-7)
8. See Appendix A for a glossary of hazard endpoint acronyms [↑](#footnote-ref-8)
9. See Appendix B for alternative GreenScreen Hazard Summary Table (Classification presented by exposure route) [↑](#footnote-ref-9)
10. For inorganic chemicals only, see GreenScreen Guidance V1.2 Section 14.4. (Exceptions for Persistence) [↑](#footnote-ref-10)
11. For Systemic Toxicity and Neurotoxicity, repeated exposure data are preferred. Lack of single exposure data is not a Data Gap when repeated exposure data are available. In that case, lack of single exposure data may be represented as NA instead of DG. See GreenScreen Guidance V1.2 Section 9.3. [↑](#footnote-ref-11)
12. See GreenScreen Guidance V1.2 Section 13 [↑](#footnote-ref-12)
13. A moiety is a discrete chemical entity that is a constituent part or component of a substance. A moiety of concern is often the parent substance itself for organic compounds. For inorganic compounds, the moiety of concern is typically a dissociated component of the substance or a transformation product. [↑](#footnote-ref-13)
14. http://echa.europa.eu/documents/10162/dd1871fd-5cbf-4dd8-98dc-90ac4bd62017 [↑](#footnote-ref-14)
15. "Where there are only local effects, at the site of administration for the routes tested, and especially when adequate testing by other principal routes show lack of specific target organ/systemic toxicity". [↑](#footnote-ref-15)
